# Emotion, Motivation, Reasoning, and How Their Brain Systems Are Related

**DOI:** 10.3390/brainsci15050507

**Published:** 2025-05-16

**Authors:** Edmund T. Rolls

**Affiliations:** 1 Oxford Centre for Computational Neuroscience, Oxford, UK; edmund.rolls@oxcns.org; 2 Department of Computer Science, University of Warwick, Coventry CV4 7AL, UK

**Keywords:** emotion, motivation, reward, reasoning, human orbitofrontal cortex, cingulate cortex, amygdala, memory, depression, consciousness, mind-brain problem, cognition

## Abstract

A unified theory of emotion and motivation is updated in which motivational states are states in which instrumental goal-directed actions are performed to obtain anticipated rewards or avoid punishers, and emotional states are states that are elicited when the (conditioned or unconditioned) instrumental reward or punisher is or is not received. This advances our understanding of emotion and motivation, for the same set of genes and associated brain systems can define the primary or unlearned rewards and punishers such as a sweet taste or pain, and the brain systems that learn to expect rewards or punishers and that therefore produce motivational and emotional states. It is argued that instrumental actions under the control of the goal are important for emotion, because they require an intervening emotional state in which an action is learned or performed to obtain the goal, that is, the reward, or to avoid the punisher. The primate including human orbitofrontal cortex computes the reward value, and the anterior cingulate cortex is involved in learning the action to obtain the goal. In contrast, when the instrumental response is overlearned and becomes a habit with stimulus–response associations, emotional states may be less involved. In another route to output, the human orbitofrontal cortex has effective connectivity to the inferior frontal gyrus regions involved in language and provides a route for declarative reports about subjective emotional states to be produced. Reasoning brain systems provide alternative strategies to obtain rewards or avoid punishers and can provide different goals for action compared to emotional systems.

## 1. Introduction and Aims

When studying emotion and its brain mechanisms, it is important to have a useful definition of emotion. The investigation of emotion and its brain mechanisms seemed to take a big step forward when an operational definition of emotion was proposed [1,2], developing earlier thinking [3,4], that emotions can be defined as states elicited by instrumental reinforcers. This definition helped many researchers to focus instead of on a previously poorly defined concept of what emotion might be, to instead study brain systems that process instrumental reinforcers, that is, rewards and punishers. Rolls’ theory of emotion [1,2,5,6,7] has led to many advances in understanding the brain systems involved in emotion and some of its disorders including depression, as described in those papers and books, with many recent advances [8,9,10,11].

The aim of the present paper is to focus on the definition of emotion (rather than on the neuroscience which is covered in detail elsewhere [8] rather than here) so that this approach can be compared to other approaches to emotion, but importantly, to extend the theory of emotion, to show how it leads to a theory of motivation, and then to show how both emotion and motivation are related to cognition and reasoning. This paper is a contribution to the Special Issue of *Brain Sciences* (2025) on ‘Defining Emotion: A Collection of Current Models’, in which different approaches to defining and understanding emotion are presented and can be compared.

In presenting my theory of emotion and motivation here, there is inevitably some textual overlap with previous descriptions of the theory, for example, in Rolls (2023) [8], but it is useful to present the approach and theory here so that it can be compared with other approaches and also developed further here. This article borrows heavily from my previous work [8,12], including sections of text and figures, which are reused here with appropriate citation and context. I emphasize that in this paper several key new areas are further developed, including the following. One is the distinction between goal-directed instrumental action, which is related to emotion, and habit-based instrumental action, which is much less related to emotion (Section 2.1, Section 2.2, Section 4.2.2 and Section 4.2.7). A second is elaboration of the different routes to action that include routes to reasoning systems that may be important in emotion beyond the goal-based action–outcome system (Section 2.2 and Section 5.4.3 and the updated Figure 3). A third is the direct comparison of a goal-directed instrumental action system which requires an intervening emotion-related state between the stimulus and the action, which is related to emotion, with systems related to classically conditioned responses such as autonomic responses and freezing behaviour, in which the amygdala has been implicated, and which are much less important in human emotion (Section 2.1 and Section 3.5). A fourth is the theory of motivation as anticipated reward/punishment states and how motivation relates to emotion as states elicited when the reward/punisher is received (Section 4.1, and including the new Figure 5). A fifth is the relation between the emotion systems and the reasoning systems (Section 2.1 and Section 5.4). A sixth is elaboration of why goal-directed instrumental action is important in understanding emotion in the framework in which a first stage of learning is stimulus–reward learning to produce an emotional state, and a second stage is instrumental action–reward outcome learning (Section 2.2). A seventh development is the importance of reward-specific satiety in emotion and reward-specific motivation (Section 3.2). An eighth development is emphasis that in Rolls’ theory of emotion, predictions are made, and errors in the predictions are corrected, so it is a model-based theory (Section 3.2). A ninth development is that in the context of a theory of declarative, conscious, emotional feelings, the connectional route between the lateral orbitofrontal cortex and language regions is emphasized (Section 5.4.3). Indeed, a key issue is which brain systems are involved in subjective conscious emotional feelings. An approach that is utilized is computational neuroscience, which asks what computations are performed by each brain region, and how they are performed [12,13,14], and this approach helps in the study of emotion and motivation by helping to specify what needs to be computed by each part of the brain and how [12,14]. A 10th development is comparisons of Rolls’ theory of motivation with other theories of motivation (Section 7). An 11th point is that new comparisons are made with Barrett’s approach to emotion (Section 6.4).

## 2. A Theory of Emotion

First, a definition and theory of emotion is provided. Part of the definition is about the functions of emotion, for they are fundamental to understanding what emotions are and how they evolved.

### 2.1. A Definition and Theory of Emotion

Emotions can usefully be defined (operationally) as states elicited by the presentation, termination, or omission of rewards and punishers which have particular functions [1,2,6,7,8,15]. A reward is anything for which an animal (which includes humans) will work. A punisher is anything that an animal will escape from or avoid. Both are instrumental reinforcers in that humans and most other animals will perform arbitrary actions to obtain the reward or avoid the goal.

**Figure 1:**
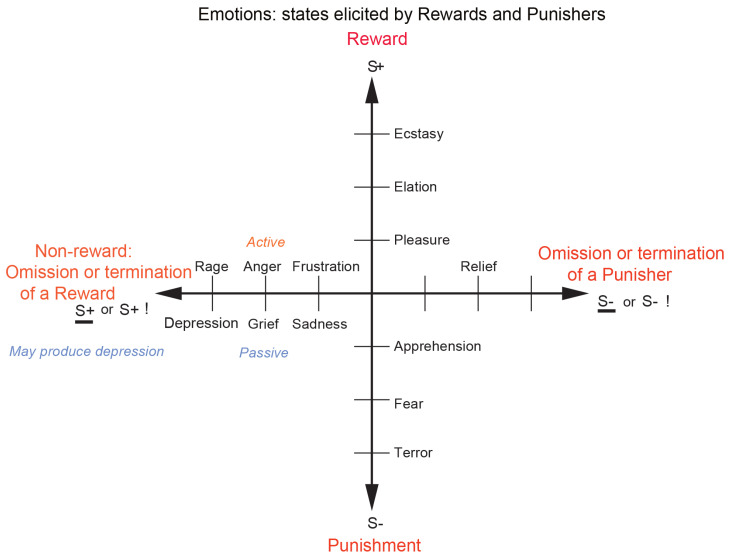
Some of the emotions associated with different reinforcement contingencies for instrumental reinforcers are indicated. Intensity increases away from the centre of the diagram, on a continuous scale. The classification scheme created by the different reinforcement contingencies consists with respect to the outcome of (1) the delivery of a reward (S+), (2) the delivery of a punisher (S−), (3) the omission of a reward (S+) (extinction) or the termination of a reward (S+!) (time out), and (4) the omission of a punisher (S−) (avoidance) or the termination of a punisher (S−!) (escape). Note that the vertical axis describes emotions associated with the delivery of a reward (up) or punisher (down). The horizontal axis describes emotions associated with the non-delivery of an expected reward (left) or the non-delivery of an expected punisher (right). For the contingency of non-reward (horizontal axis, left) different emotions can arise depending on whether an active action is possible to respond to the non-reward, or whether no action is possible, which is labelled as the passive condition. In the passive condition, non-reward may produce depression. Frustration could include disappointment. The diagram summarizes emotions that might result for one reinforcer as a result of different contingencies. Every separate reinforcer has the potential to operate according to contingencies such as these. This diagram does not imply a dimensional theory of emotion but shows the types of emotional state that might be produced by a specific reinforcer. Each different reinforcer will produce different emotional states, but the contingencies will operate as shown to produce different specific emotional states for each different reinforcer. (Modified from [12] Rolls, E. T. (2023) Brain Computations and Connectivity. Oxford University Press: Oxford. Open Access CC BY-NC-ND 4.0).

As shown in Figure 1, different reward/punishment contingencies are associated with different types of emotion. An example of an emotion associated with a reward might be the happiness produced by being given a particular reward, such as a pleasant touch, praise, or winning a large sum of money. An example of an emotion produced by a punisher might be fear produced by the sound of a rapidly approaching bus or the sight of an angry expression on someone’s face. We will work to avoid such punishing stimuli. An example of an emotion produced by the omission, termination, or loss of a reward is frustration or anger (if some action can be taken), or sadness (if no action can be taken). An example of an emotion produced by the omission or termination of a punisher (such as the removal of a painful stimulus or sailing out of danger) would be relief. These examples indicate how emotions can be produced by the delivery, omission, or termination of rewarding or punishing stimuli and go some way to indicate how different emotions could be produced and classified in terms of the rewards and punishers received, omitted, or terminated. Figure 1 summarizes some of the emotions associated with the delivery of a reward, or punisher, or a stimulus associated with them, or with the omission of a reward or punisher.

I consider elsewhere a more formal definition than rewards or punishers, in which the concept of reinforcers is introduced, and it is shown that emotions can be usefully seen as states produced by instrumental reinforcing stimuli [6]. Instrumental reinforcers are stimuli which, if their occurrence, termination, or omission is made contingent upon the making of a behavioural response (action), alter the probability of the future emission of that response (action) [16].

Some stimuli are unlearned (innate), “primary”, reinforcers (e.g., the taste of food if the animal is hungry, pain, or a pleasant touch). Some examples of primary reinforcers are shown in Table 1 [6]. There may be in the order of 100 such primary reinforcers, each specified by different genes [6]. Each primary reinforcer can produce a different type of affective state; for example, the taste of a pleasant sweet or sweet/fat texture food such as ice cream is very different from the feel of a pleasant touch vs. pain, and they are all in turn very different from attraction to or love for someone. Thus, different types of affective state are produced by each different primary reinforcer, and the reinforcement contingencies shown in Figure 1 apply to each of these primary reinforcers. For example, not receiving ice cream is very different emotionally from not receiving pleasant touch.

Other stimuli may become reinforcing by associative learning because of their association with such primary reinforcers, thereby becoming “secondary reinforcers”. An example might be the sight of a painful stimulus. Brain systems that learn and unlearn these associations between stimuli or events in the environment and reinforcers are important in understanding the neuroscience and neurology of emotions, as we will see below.

This foundation has been developed by Rolls (2014) [6] to show how a very wide range of emotions can be accounted for, as a result of the operation of a number of factors, including the following:

1. The *reinforcement contingency* (e.g., whether a reward or punishment is given or withheld) (see Figure 1).
2. The *intensity* of the reinforcer (see Figure 1).
3. Any environmental stimulus might have a *number of different reinforcement associations*. (For example, a stimulus might be associated both with the presentation of a reward and a punisher, allowing states such as conflict and guilt to arise.)
4. Emotions elicited by stimuli associated with *different primary reinforcers* will be different, as described above, for different primary reinforcers, each of which will produce different affective states, as shown in Table 1.
5. Emotions elicited by *different secondary reinforcing stimuli* will be different from each other (even if the primary reinforcer is similar). For example, the same touch to the arm but by different people might give rise to very different emotions. Cognitive states and semantic knowledge can contribute to emotion in these ways, as well as in other ways that might arise because, for example, of reasoning in the rational brain system.
6. The emotion elicited can depend on whether an *active or passive behavioural response* is possible. (For example, if an active behavioural response can occur due to the omission of a positive reinforcer, then anger might be produced, but if only passive behaviour is possible, then sadness, depression, or grief might occur (see Figure 1)).

By combining these six factors, it is possible to account for a very wide range of emotions, as described by Rolls [6]. This is important: the range of emotions that can be accounted for in this way is enormous because each emotional state is distinguished by the reinforcement contingency, the particular primary reinforcer, the particular secondary reinforcer, the particular combination of reinforcers, the intensity of each reinforcer, etc., as specified above [6], and is not limited [18]. It is also worth noting that emotions can be produced just as much by the recall of reinforcing events as by external reinforcing stimuli; and that cognitive processing (whether conscious or not) is important in many emotions, for very complex cognitive processing may be required to determine whether or not environmental events are reinforcing. Indeed, emotions normally consist of cognitive processing that analyses the stimulus, and then determines its reinforcing valence, and then elicits an affective (emotional) state or longer-term mood change if the valence is positive or negative. I note that a mood or affective state may occur in the absence of an external stimulus, as in some types of depression, but that normally the mood or affective state is produced by an external stimulus, with the whole process of stimulus representation, evaluation in terms of reward or punishment, and the resulting mood or affect being referred to as emotion [6].

Emotion-related learning in this framework has two stages [cf. 4]. In the first stage, an association is learned between a stimulus such as the sight of food and a primary (unlearned) reward, such as the taste of food. Another example would be the sight of a person associated with a pleasant touch (a primary reinforcer). This is a stimulus–reward type of learning in which the associated stimulus and the primary reinforcer are both stimuli. In the brain, the orbitofrontal cortex is involved in this type of learning and in rapidly reversing the learning if the contingency switches [8]. The result of the learning is that an emotional state is produced by the conditioned stimulus, such as the sight of food or the sight of the person. The emotional state might be pleasure produced by the sight of the food or the person. That is an “expected value” signal [8,12]. And consistently, it is a property of this emotional state that it is motivating. The sight of the food may make us wish to work to obtain the food or work to be with the person. And that is the second stage of the process: learning what action to take to obtain the food reward (perhaps picking a fruit from a tree, paying in a shop for the food, or travelling to be with the person). That is action–outcome learning, that is, learning the action that will lead to obtaining the desired outcome of obtaining the reward or avoiding the punisher. In the brain, the anterior cingulate cortex is involved in action–outcome learning, for which it receives action-related information from premotor cortical regions, and expected value and reward outcome information from the orbitofrontal cortex [8,19,20].

A key issue is that emotions occur strongly when the instrumental action is under the control of the goal or reward, and the emotions are typically much weaker after instrumental learning has developed to the stage where the behaviour becomes a well-learned habit, essentially a stimulus–response type of behaviour, rather than having the reward value being processed and producing an intervening emotional state that helps to guide actions being performed in order to obtain the reward or avoid the punisher. The background here is that early on in learning (including when no action has yet been learned to obtain the reward or avoid the punisher), then if the reward is devalued outside the task (for example by feeding the food to satiety), no action will be performed in the task to obtain the reward. But if the instrumental action has become highly learned, it becomes a habit or stimulus–response type of behaviour in that devaluation of the reward makes no difference, and the response is still elicited by the stimulus [21,22]. In this habit phase, there is no need for any intervening emotional state between the stimulus and the response which now becomes automated as a stimulus–response habit, and correspondingly, the individual can perform the response with very few correlates of emotion such as a raised heart rate. (Indeed, reference to autonomic correlates of emotional states reminds one of the traditional view that emotions are something that may be occurring when the heart is responding and to the use of a heart symbol for love.) The brain system that implements this habit level type of automated stimulus–response behaviour is the basal ganglia [12].

*The result of this point is that the definition of emotion needs to refer to states elicited by instrumental reinforcers when the behaviour is under the control of the reward or punisher, the goal value, and not when an instrumental habit has been set up and is being used.*

The implication of what has been presented so far is that emotions are intervening states elicited by an instrumental reinforcer that is providing the goal for an action before a stimulus–response habit link has been set up. This helps to explain why classically conditioned responses, such as autonomic responses or freezing behaviour, do not provide a good model of emotion. In classical (Pavlovian) conditioning, an association between a conditioned stimulus such as Pavlov’s bell and the delivery of food elicits behaviour such as salivation, but no intervening state is needed: the sound of the bell can be directly associated with the taste of food or salivation, and the bell then produces salivation. There is no goal for an action that is needed as there are no instrumental actions involved, and no goals for action need to be represented to motivate an action to obtain the goal. This is extremely telling, for although there has been a massive research effort to use classically conditioned responses such as autonomic responses to a sound associated with a shock as a model for emotion and the amygdala was identified as a key brain system involved in emotion [23,24,25,26,27,28,29,30,31,32], it has now been realized that although amygdala damage may impair some classically conditioned responses to stimuli, this damage leaves most emotional behaviour and emotional feelings hardly changed in humans [33,34,35,36,37,38], so classical conditioning has not be a very good model of human emotion and emotional feelings.

The subjective feelings of emotions are part of the much larger problem of consciousness [39]. The brain bases of subjective experience are a topic of considerable current interest, not only with higher-order thought (HOT) theories [40,41] but also with the higher-order syntactic thought (HOST) theory of consciousness [6,7,13,39,42,43], which is more computationally specific and addresses the adaptive value of the type of processing related to consciousness; and a point made here is that the orbitofrontal cortex is at least on the route to human subjective experiences of emotion and affective value (see Section 5.4.3).

### 2.2. The Functions of Emotions

Part of the definition or description of emotions must refer to the functions that emotion performs. The functions of emotion are considered now.

**Figure 2:**
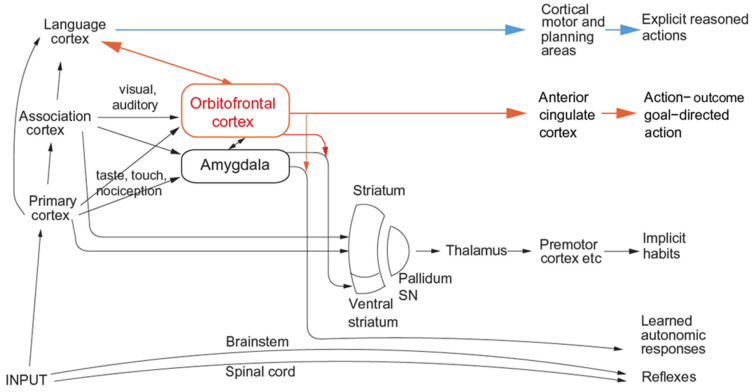
Multiple routes to the initiation of actions and responses to rewarding and punishing stimuli in primates including humans. The lowest (spinal cord and brainstem) levels in the hierarchy are involved in reflexes, including, for example, reflex withdrawal of a limb to a nociceptive stimulus, and unlearned autonomic responses. The second level in the hierarchy involves associative learning in the amygdala and orbitofrontal cortex between primary reinforcers such as taste, touch and nociceptive stimuli and neutral stimuli such as visual and auditory stimuli from association cortex (e.g., inferior temporal visual cortex) to produce learned autonomic and some other behavioural responses such as approach. The anteroventral viscero-autonomic insula may be one link from the orbitofrontal cortex to autonomic output. A third level in the hierarchy is the route from the orbitofrontal cortex and amygdala via the basal ganglia especially the ventral striatum to produce implicit stimulus-response habits. A fourth level in the hierarchy important in emotion is from the orbitofrontal cortex to the anterior cingulate cortex for actions that depend on the value of the goal in action-outcome learning. For this route, the orbitofrontal cortex implements stimulus-reinforcer association learning, and the anterior cingulate cortex action-outcome learning (where the outcome refers to receiving or not receiving a reward or punisher). A fifth level in the hierarchy is from the orbitofrontal cortex (and much less the amygdala [61]) via multiple step reasoning systems involving syntax and language. Processing at this fifth level may be related to explicit conscious declarative states. The fifth level may also allow some top-down control of emotion-related states in the orbitofrontal cortex by the explicit processing system. Pallidum/SN—the globus pallidus and substantia nigra. (Modified from [12] Rolls, E. T. (2023) Brain Computations and Connectivity. Oxford University Press: Oxford. Open Access CC BY-NC-ND 4.0).

**Figure 3:**
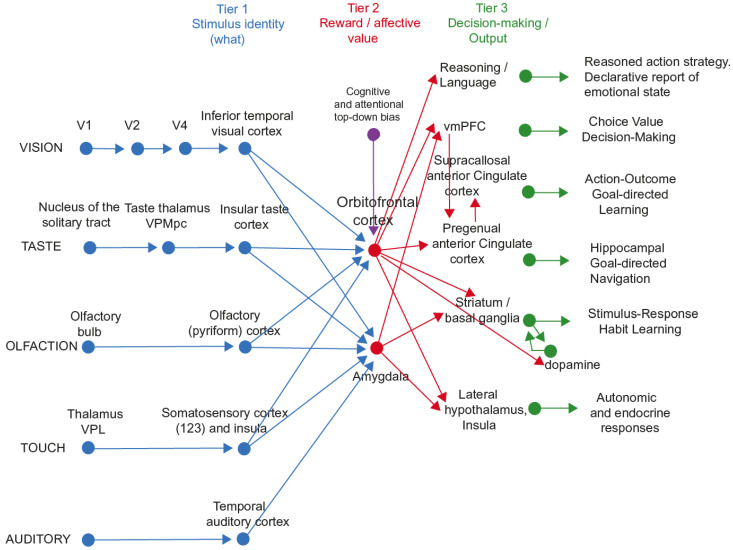
The system-level organization of the brain for emotion in primates including humans. In Tier 1, representations are built from visual, taste, olfactory, and tactile stimuli that are independent of reward value and therefore of emotion. In Tier 2, reward value and emotion are represented. A pathway for top-down attentional and cognitive modulation of emotion from, for example, the prefrontal cortex [62] is shown in purple. In Tier 3, actions are learned in the supracallosal (or dorsal) anterior cingulate cortex to obtain the reward values signalled by the orbitofrontal cortex and amygdala that are relayed in part via the pregenual anterior cingulate cortex and vmPFC. Decisions between stimuli of different reward values can be taken in the ventromedial prefrontal cortex, vmPFC. In Tier 3, orbitofrontal cortex inputs to the reasoning/language systems enable affective value to be incorporated and reported. In Tier 3, stimulus–response habits can also be produced using reinforcement learning. In Tier 3, autonomic responses can also be produced to emotion-provoking stimuli. Auditory inputs also reach the amygdala. V1—primary visual (striate) cortex; V2 and V4—further cortical visual areas. PFC—prefrontal cortex. The Medial PFC area 10 is part of the ventromedial prefrontal cortex (vmPFC). VPL—ventro-postero-lateral nucleus of the thalamus, which conveys somatosensory information to the primary somatosensory cortex (areas 1, 2, and 3). VPMpc—ventro-postero-medial nucleus pars parvocellularis of the thalamus, which conveys taste information to the primary taste cortex. (Modified from [12] Rolls, E. T. (2023) Brain Computations and Connectivity. Oxford University Press: Oxford. Open Access CC BY-NC-ND 4.0).

The most important function of emotion is as part of the processes of learning goal-directed actions to obtain rewards or avoid punishers, as outlined above. The first process is stimulus–reinforcer association learning; emotional states are produced as a result [6]. An example might be learning that the sight of a person is associated with rewards, which might produce the emotion of happiness. This process is implemented in structures such as the orbitofrontal cortex and amygdala (Figure 2 and Figure 3) [6,8,12,44,45].

The second process is instrumental learning of an action made to approach and obtain the reward (an outcome of the action) or to avoid or escape from the punisher (an outcome). This is action–outcome learning and involves brain regions such as the anterior cingulate cortex when the actions are being guided by the goals [6,7,12,14,19,46,47]. Emotion is an integral part of this, for it is the state elicited in the first stage by stimuli that are decoded as rewards or punishers [6]. The behaviour is under control of the reward value of the goal in that if the reward is devalued, for example by feeding a food until satiety is reached, then on the very next occasion that the stimulus (the food) is offered, no action will be performed to try to obtain it, as described above [6].

The striatum, the rest of the basal ganglia, and the dopamine system can become involved when the behaviour becomes automatic and habit-based and uses stimulus–response associations (Figure 2 and Figure 3). In this situation, very little emotion may be elicited by the stimulus, as the behaviour has now become automated as a stimulus–response habit. For this type of learning, if the reward is devalued outside the situation, then the very next time that the stimulus is offered, the automated response is likely to be performed, providing evidence that the behaviour is no longer being guided by the reward value of the stimulus, which is the goal. The dopamine system is involved in this type of rather slow habit-based learning by providing an error signal to the striatum which implements this type of habit learning [48,49,50]. The dopamine system probably receives its inputs from the orbitofrontal cortex [20,51]. These brain systems are considered further elsewhere [8,12].

Other functions of emotion include the elicitation of autonomic responses via pathways, for example, from the amygdala and also the orbitofrontal cortex to the anteroventral visceral/autonomic insula and to the subgenual cingulate cortex [6,15,19,23,24,25,52,53,54,55,56]. That function is useful for preparing the body for action. Another classically conditionable response is freezing, and that may also involve the amygdala in rodents [24,25]. Comparable classically conditioned responses can occur in humans [57,58,59,60].

The multiplicity of different types of behavioural responses that can be elicited by emotion-provoking stimuli is shown by the different neural routes involved, as illustrated for primates including humans in Figure 2 and Figure 3. An important point made by Figure 2 and Figure 3 is that there are multiple routes to output including to action that can be produced by stimuli that produce emotional states. Here, emotional states are the states elicited by reward and punishing/non-reward stimuli, as illustrated in Figure 1.

The multiple routes are organized in a set of hierarchies, with each level in the system added later in evolution but with all levels left in operation over the course of evolution [13]. The result of this is that a response such as an autonomic response to a stimulus that happens to be rewarding might be produced by only the lower levels of the system operating without necessarily the highest, explicit, levels being involved. The lowest levels in the hierarchy illustrated in Figure 2 and Figure 3 are involved in reflexes, including, for example, reflex withdrawal of a limb to a nociceptive stimulus and autonomic responses.

The second level in the hierarchy (Figure 2 and Figure 3) can produce learned autonomic and some other behavioural responses to, for example, a previously neutral visual or auditory stimulus after it has been paired with a nociceptive stimulus or with a good taste stimulus. This route involves stimulus–reinforcer learning in the amygdala and orbitofrontal cortex.

A third level in the hierarchy shown in Figure 2 and Figure 3 is the route from the orbitofrontal cortex and amygdala via the basal ganglia especially the ventral striatum to produce implicit stimulus–response habits.

A fourth level in the hierarchy that is important in emotion is from the orbitofrontal cortex to the anterior cingulate cortex for goal-directed action. The emotional states implemented at this level may not necessarily be conscious.

A fifth level in the hierarchy shown in Figure 2 and Figure 3 is from the orbitofrontal cortex (and much less the amygdala [61]) via multiple step reasoning systems involving syntax and language, which can be associated with explicit conscious states (especially if a higher-order syntactic thought system for correcting lower-order thoughts is involved [6,12,39,63], see Section 5.4). A key update to my theory of emotion is that although goal-directed goal-dependent instrumental actions are key to understanding emotion, it is possible that all of that could take place without the fifth layer in the hierarchy, the reasoning system, being involved. The syntactic reasoning system may be of very great adaptive value in humans and other animals with such systems by enabling these individuals to reason about their emotional states and choose plans based on multi-step syntactic reasoning to maximize their rewards (and minimize their punishers), which are represented by their emotional states (the intervening variables referred to above). It is suggested below that this type of computation, reasoning, and especially correcting reasoning by higher-order syntactic thoughts, and using that reasoning system to deal with emotional states as defined above that are elicited by instrumental reinforcers, may be closely related to conscious emotional feelings, though that is a plausibility argument only (see Section 5.4.3 on conscious emotional feelings).

**Figure 4:**
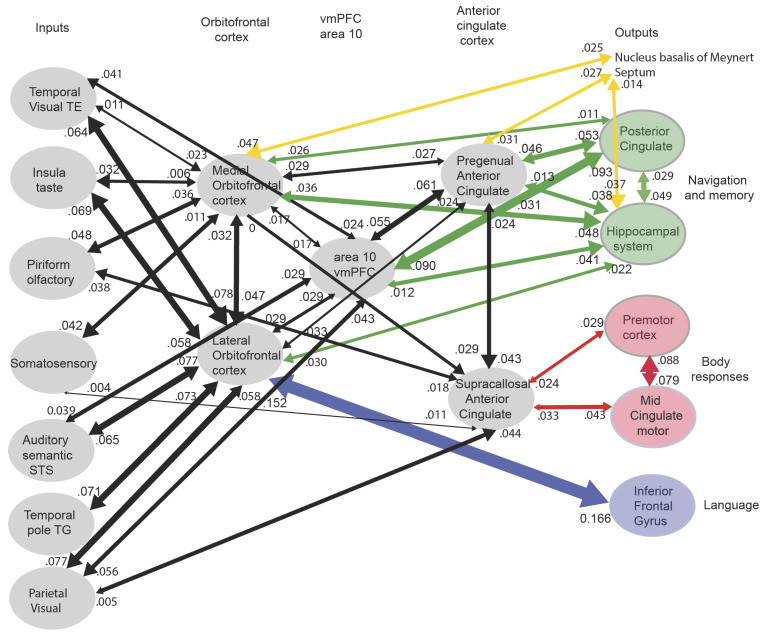
Effective connectivity of the human orbitofrontal cortex, vmPFC, and anterior cingulate cortex shown in the middle, with inputs on the left and outputs on the right. The effective connectivity was measured in 171 participants imaged at 7T by the Human Connectome Project and was measured between the 360 cortical regions in the HCP-multimodal parcellation atlas [65], with subcortical regions using the HCPex atlas [66]. Effective connectivity measures the effect in each direction between every pair of cortical regions, uses time delays to assess the directionality using a Hopf computational model which integrates the dynamics of Stuart–Landau oscillators in each cortical region, has a maximal value of 0.2, and is described in detail elsewhere [20,67,68]. The width of the arrows is proportional to the effective connectivity in the highest direction, and the size of the arrowheads reflects the strength of the effective connectivity in each direction. The effective connectivities shown by the numbers are for the strongest link where more than one link between regions applies for a group of brain regions. Effective connectivities with hippocampal memory system regions are shown in green, with premotor/mid-cingulate regions in red, the inferior prefrontal language system in blue, and the basal forebrain nuclei of Meynert in yellow which contain cholinergic neurons that project to the neocortex and to the septal nuclei which contain cholinergic neurons that project to the hippocampus. The Somatosensory regions include 5 and parietal PF and PFop, which also connect to the pregenual anterior cingulate but are not shown for clarity; the Parietal regions include visual parietal regions 7, PGi, and PFm. The inputs to the hippocampal system from the orbitofrontal cortex and connected regions are largely via the perirhinal and entorhinal cortex. (From [12] Rolls, E. T. (2023) Brain Computations and Connectivity. Oxford University Press: Oxford. Open Access CC BY-NC-ND 4.0).

The connectivity of the human orbitofrontal cortex (Figure 4) shows that these routes are present in humans, and emphasizes that there are major outputs from the orbitofrontal cortex reward and punisher systems to the hippocampal system that allow rewards and punishers to be stored as part of episodic memories and also to influence memory consolidation [20,64].

It is emphasized that each of these types of output have adaptive value in preparing individuals to deal physiologically and behaviourally with what may generally be described as emotion-provoking events.

## 3. A Framework for Understanding the Neuroscience of Emotion in Humans and Other Primates

Given that the focus of this paper is on Rolls’ theory of emotion, motivation, and their relationships to cognition and reasoning, the brain mechanisms of emotion are not considered in detail here, but they are considered elsewhere [6,7,8,10,12,19,20,52,69,70,71], with a key paper by Rolls (2023) [8]. However, highlights and additional points especially relevant to the consideration of emotions are summarized here.

### 3.1. A Framework for the Neuroscience of Emotion

A framework is shown in Figure 3, and it is built on evidence from neuronal recordings in macaques, fMRI, and the effects of brain damage in humans [6,7,8,10,12,19]. Part of the evidence for what is shown in Figure 3 comes from reward devaluation, in which when the reward value is changed, for example by feeding to satiety, neural responses to stimuli are little affected in Tier 1 but decrease to zero in Tier 2. Part of the evidence comes from the learning of associations between stimuli and reward value, which occurs mainly in Tier 2. Part of the evidence comes from the effects of brain damage on emotion, which occur primarily after damage to the orbitofrontal cortex and amygdala in Tier 2, and the anterior cingulate cortex in Tier 3 [72].

A key point about Figure 2 and Figure 3 is that it is especially the orbitofrontal cortex and much less the amygdala that has connectivity to reasoning/language systems in the human brain, and this is part of the human brain organization that may be closely related to declarative reports about emotional stimuli and perhaps to conscious emotional feelings. What is relevant in this respect is that the subjective conscious-rated pleasantness of many rewarding stimuli is linearly related to activations in the orbitofrontal cortex [8,12].

The organization of reward value processing and therefore emotion in the rodent brain is very different [12,52], and that is described elsewhere [8,12]. For this reason, emphasis here is placed on systems-level investigations in primates and humans, for there is evidence that many cortical systems operate so differently in rodents [12,73]. Some of these differences are set out in section 19.10 of *Brain Computations and Connectivity* [12].

### 3.2. The Human Medial Orbitofrontal Cortex Represents Reward Value

The primate including human orbitofrontal cortex is the first stage of cortical processing that represents reward value (red in Tier 2 in Figure 3; see also Figure 4), as shown by reward devaluation and visual discrimination reversal learning experiments [8,11,12,52].

An important principle of emotion is the discovery of reward-specific satiety or sensory-specific satiety which is implemented in the orbitofrontal cortex [12,74,75,76,77], and it has enormous implications, for it has the evolutionary adaptive value that behaviour switches from one reward to another. This ensures, for example, that a wide range of nutrients will be ingested [78] and more generally tends to promote reproductive success for the genes in that a wide range of possible rewards will be explored [6,7,12]. Sensory-specific satiety is thus a key factor in emotion and the choice of what reward is the current goal for action [74].

Another important concept from neuroscience for understanding emotion is that predictions are made of outcomes in that expected value is represented in the orbitofrontal cortex, and in that if the reward values of two stimuli are reversed, non-reward or error neurons in the orbitofrontal cortex are activated, and behavioural reversal to choose the previously unrewarded visual stimulus implemented in the primate and human orbitofrontal cortex occurs as soon as non-reward is received for one stimulus, providing evidence for model-based operation of the primate including the human emotion system [79,80,81,82]. This is very appropriate for primates including humans who in social situations may benefit from being very responsive to non-reward vs. reward signals, and may not occur in rodents [12,52,83].

Another important concept is that the orbitofrontal cortex contains representations for many different types of rewarding stimuli, showing how the orbitofrontal cortex is involved in a wide range of emotions including those involved in social behaviour. For example, the macaque orbitofrontal cortex contains visual neurons that reflect face expression and face identity (both necessary to decode the reward/punishment value of an individual) [84]. Information about face expression and movements important in social communication probably reaches the orbitofrontal cortex from neurons we discovered in the cortex in the macaque superior temporal sulcus that respond to these stimuli [75,85,86] in what is a region now accepted as important for decoding visual stimuli relevant to social behaviour [87,88]. There is consistent evidence from neuroimaging [12,89,90,91,92,93], which allows the types of reward to be extended to include monetary reward [94,95,96], face expressions [93], and face beauty [97].

Another important concept is that the orbitofrontal cortex is implicated in human subjectively reported emotional states in that activations of the medial orbitofrontal cortex are linearly related to the subjective (conscious) pleasantness of stimuli [12,44,52]. These reward-related effects are found for odours [98], flavours [89,90], a pleasant touch [99,100], monetary rewards [94,95], and amphetamines [101]. A causal role in these subjective emotional states is that humans with orbitofrontal cortex lesions have reduced subjective emotional feelings [102,103], as well as difficulty in identifying face and voice emotion-related expressions, which are important for emotional and social behaviour [102,104].

### 3.3. The Human Lateral Orbitofrontal Cortex Represents Punishers and Non-Rewards and Is Involved in Changing Emotional Behaviour

The roles of different instrumental contingencies in emotion indicated in Figure 1 are supported by discoveries that not obtaining an expected reward, ‘non-reward’, is represented in the orbitofrontal cortex which as we have seen is involved in emotion. In particular, the macaque orbitofrontal cortex has neurons that respond when an expected reward is not received [79], and these have been termed *non-reward neurons* [6,8,11,12,52]. These neurons do not respond to expected punishers (e.g., a discriminative stimulus for mildly aversive saline [79]), but other neurons do respond to expected punishers [80], showing that non-reward and punishment are represented by different neurons in the orbitofrontal cortex [8,12,52].

Corresponding to this, the human lateral orbitofrontal cortex is activated when a reward is not obtained in a visual discrimination reversal task [93], when money is not received in a monetary reward task [94,95], and in a one-trial reward reversal task [96]. Further, the human lateral orbitofrontal cortex is also activated by punishing, subjectively unpleasant, stimuli [11,12,44,52].

These discoveries show that one way in which the orbitofrontal cortex is involved in emotion and decision-making is by representing rewards, punishers, and errors made during decision-making, consistent with what Figure 1 indicates are key contingencies that are involved in emotion. This is supported by the problems that orbitofrontal cortex damage produces in decision-making, which include failing to respond correctly to non-rewards [12,72]. This type of flexibility of behaviour is important in primate including human social interactions and emotional behaviour, and indeed, many of the effects of damage to the human orbitofrontal cortex, including the difficulty in responding appropriately to the changed circumstances of the patient and the changed personality including impulsivity, can be related to these impairments in responding to non-rewards and punishers [7,10,11,12,52,72,103,105,106,107].

### 3.4. The Ventromedial Prefrontal Cortex and Reward-Related Decision-Making

The ventromedial prefrontal cortex (vmPFC), which receives inputs from the orbitofrontal cortex and has outputs to the anterior cingulate cortex [20,108] (Figure 3 and Figure 4), has long been implicated in reward-related decision-making [45,109,110,111,112] and has the signature of a decision-making region of increasing its activation in proportion to the difference in the decision variables, which correlates with decision confidence [12,52,113,114].

### 3.5. The Amygdala

The amygdala in rodents, in which the orbitofrontal cortex is much less developed than in primates [73,115], has been implicated in emotion-related responses such as conditioned autonomic responses, conditioned freezing behaviour, cortical arousal, and learned incentive effects in fear conditioning in which an auditory tone is associated with foot shock [25,26,116]. Synaptic modification in the amygdala is implicated in the learning of these types of responses [23,24,27,31,32,117,118].

However, classical conditioning can be implemented by stimulus–response (or possibly stimulus–stimulus) associations between the tone and the shock used in these investigations and does not involve an intervening emotional state as in the goal-directed instrumental learning in Rolls’ theory of emotion. Consistent with the view that classical conditioning may not be a good model for emotion, the effects of damage to the human amygdala indicate that it is rather little involved in subjective emotional experience [33,35,36,38,57,61,119,120,121,122,123,124,125], in contrast to the orbitofrontal cortex [52,72,102,103,104,105,126,127].

Part of the basis for this may be that the amygdala has many subcortical outputs in rodents [25] and in humans [128], and in humans it has much less connectivity back to the neocortex including language areas than the orbitofrontal cortex [61] (Figure 2 and Figure 3). Accordingly it is proposed that the human amygdala is involved primarily in autonomic and conditioned responses via brainstem connectivity, rather than in reported (declarative) emotion [61].

LeDoux’s conundrum is as follows: if not the amygdala for subjective emotional experience, then what [125]? My answer is as follows: the human orbitofrontal cortex is the key brain region involved in subjective emotion in humans and other primates [6,12,52,61,72].

The problem of over-interpreting the role of the amygdala in emotion was that rodent studies showed that some responses such as classically conditioned autonomic responses and freezing are elicited by the amygdala with its outputs to brainstem systems, and it was inferred that the amygdala is therefore involved in emotion in the way that it is experienced by humans [23,25,26,116]. It turned out later that humans with amygdala damage had similar response-related changes but little impairment in subjectively experienced and reported emotions [35,36,57,61,119,121]. It is important, therefore, it is argued to not infer subjective reported emotional states in humans from responses such as conditioned autonomic and freezing responses [8,61]. This dissociation of autonomic response systems from subjectively felt and reported emotions in humans is further evidence against the James–Lange theory of emotion and the related somatic marker hypothesis of Damasio [129,130] (see Rolls [6] and Section 6.1).

A comparison of my theory of emotion with other theories of emotion is provided in Section 6, but to maintain the continuity of the argument presented in this paper, I now move to relate Rolls’ theory of emotion to my theory of motivation.

## 4. A Theory of Motivation and Brain Systems That Implement Motivation

I now describe and extend Rolls’ theory of motivation [8], which complements and utilizes many of the same brain systems as Rolls’ theory of emotion.

### 4.1. The Outline of a Theory of Motivation

First, the essence of Rolls’ approach to motivation is described. **My definition of motivation is that motivational states are states that are present when rewards and punishers, that is, instrumental reinforcers, are the goals for action** [6,8,131]. A reward is anything for which an animal (and this includes humans) will work. A punisher is anything that an animal will work to escape or avoid, or that will suppress actions on which it is contingent [6]. The force of ‘instrumental’ in this definition is that the motivational states are seen as defining the goals for arbitrary behavioural actions made to obtain the instrumental reinforcer, which is the goal for the action. This is very different from classical conditioning, in which a response, typically autonomic, may be elicited to a stimulus without any need for an intervening state [6] (see above). The motivational states (such as hunger) modulate the reward value/goal value of instrumental reinforcers that have particular functions [6,131]. It is important in this definition that the reward values of potential goals are regulated appropriately, with, for example, factors such as plasma glucose, gastric distension, and absorbed food acting to control the reward value of food [5,78], and cellular and extracellular dehydration modulating the reward value of water [5,132,133,134].

An example of a motivational state might thus be a hunger state in which the animal will perform goal-directed actions to obtain the reinforcer or goal. Another example is that the omission or termination of a reward (‘extinction’ and ‘time out’, respectively) can produce a motivational state of frustration, in which the probability of the action may become reduced if no action is possible to regain the reward, or may increase if further motivated attempts are likely to lead to the reward [6,131].

**Figure 5:**
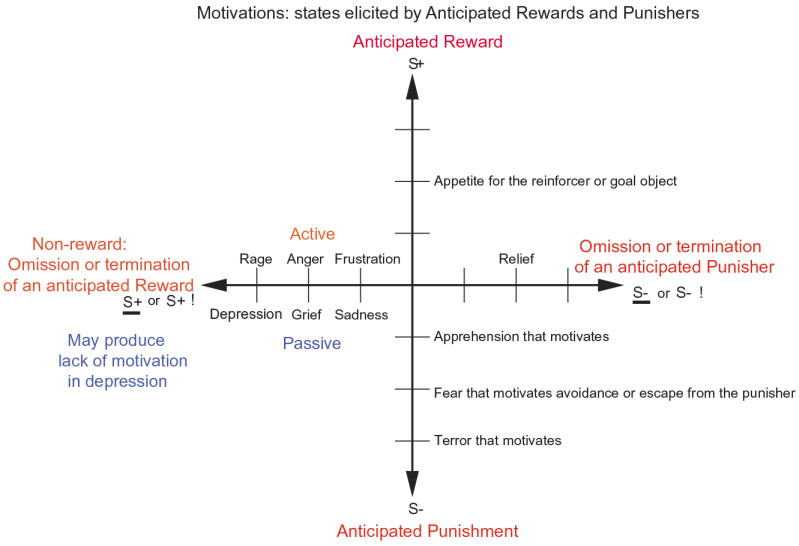
Some of the motivational states associated with different reinforcement contingencies for anticipated rewards and punishers are indicated. An anticipated reward motivates behaviour to obtain the reward which is the goal of an instrumental action; and an anticipated punisher motivates behaviour to avoid or escape from the punisher which is the goal of an instrumental action. Intensity increases away from the centre of the diagram, on a continuous scale. The classification scheme shows how different motivational states created by the different reinforcement contingencies consist of being in a state that leads to (1) wanting to perform an action to obtain an anticipated reward (S+), (2) wanting to perform an action to escape from or avoid an anticipated punisher (S−), (3) wanting to perform an action or not because of the omission of an anticipated reward (S+) (extinction) or the termination of a reward (S+!) (time out), and (4) wanting to perform an action or not because of the omission of a punisher (S−) (avoidance) or the termination of a punisher (S−!) (escape). Note that the vertical axis describes motivational states associated with the expected delivery of a reward (up) or punisher (down). The horizontal axis describes motivational states associated with the non-delivery of an expected reward (left) or the non-delivery of an expected punisher (right), and any action depends on what is possible in the environment. The diagram summarizes motivational states that might arise for one reinforcer as a result of different contingencies. Every separate reinforcer has the potential to operate according to contingencies such as these. Each different reinforcer will produce different motivational states, but the contingencies will operate as shown to produce different specific motivational states for each different reinforcer. Emotional states elicited by expected reinforcers can produce motivational states to work for an expected reward or avoid an expected punisher.

These examples show that the reinforcement contingency as well as the particular reinforcer or goal object (e.g., food, water, and aversive stimulation) lead to particular motivational states. The types of motivational states related to different reinforcement contingencies such as anticipated reward or anticipated punishment are illustrated in Figure 5. The diagram summarizes motivational states that might relate to one reinforcer as a result of different contingencies. Every separate reinforcer has the potential to operate according to contingencies such as these in an analogous way to that described above for emotional states. Each different reinforcer will produce different motivational states, but the contingencies will operate as shown to produce different specific motivational states for each different reinforcer. Thus, hunger might be present when the appetite is for the goal object of food, and thirst when the appetite is for the goal object of water. Definitions of reinforcers and of the contingencies with which they operate are elaborated by Rolls [6].

We must be clear about the difference between motivation and emotion. According to Rolls’ theory of emotion described above, emotion is the state that results from having received or not having received the (conditioned or unconditioned) instrumental reinforcer, which is the goal object [6,7,8]. In contrast, motivation is the state when the instrumental reinforcer is anticipated (Figure 5), being worked for, before the outcome stage, where the outcome is the delivery or not of the reinforcer, the reward, or the punisher. **An important attribute of this theory of motivation and emotion is that the goal objects can be the same for motivation and emotion, simplifying the biological specification, with the difference being that motivation is the phase before the outcome when the outcome is anticipated, and emotion is the phase after the conditioned or unconditioned reward or punisher has been received.** An additional property is that emotions, states occurring after the delivery or not of the reinforcer, can be motivating [6]. A good example is that if an expected reward is not obtained, then the frustrative non-reward can be motivating and make the animal (including humans) work harder to obtain the goal object [6].

As described above for emotion, reinforcers, that is, rewards or punishers, may be unlearned or **primary reinforcers** or learned, that is, secondary reinforcers. An example of a primary reinforcer is pain, which is innately a punisher. The first time a painful stimulus is ever delivered, it will be escaped from, and no learning that it is aversive is needed. Similarly, the first time a sweet taste is delivered, it acts as a positive reinforcer, so it is a primary positive reinforcer or reward. Other stimuli become reinforcing by learning because of their association with primary reinforcers, thereby becoming ‘**secondary reinforcers**’. For example, the sight of a food that regularly precedes the flavour of the food can rapidly become a secondary reinforcer. Motivational states can relate to primary or secondary (learned) reinforcers. For example, a human might want to see another individual, and that would be motivation related to a secondary (conditioned) reinforcer; and motivation might be related to wanting a primary (unconditioned) reinforcer, such as a pleasant touch.

Some examples of primary reinforcers are provided in Table 1. All of the primary reinforcers or goal objects can elicit different, specific, motivational states. As these are primary reinforcers, they are likely to be gene-specified [6].

### 4.2. Motivational States Are States That Modulate the Reward or Punishment Value of Instrumental Reinforcers and Are Different from Taxes, Approach Responses, Classical Conditioning, and Stimulus–Response Habits

#### 4.2.1. Taxes

A simple design principle is to incorporate mechanisms for taxes into the design of organisms. Taxes consist at their simplest of orientation towards stimuli in the environment, such as the bending of a plant towards light that results in maximum light collection by its photosynthetic surfaces. When just turning rather than locomotion is possible, such responses are called tropisms. With locomotion possible, as in animals, taxes include movements toward sources of nutrient and movements away from hazards such as very high temperatures. The design principle here is that animals have, through a process of natural selection, built receptors for certain dimensions of the wide range of stimuli in the environment and have linked these receptors to response mechanisms in such a way that the stimuli are approached or escaped from. This could include a single-cell organism such as *Euglena* moving towards light.

#### 4.2.2. Habit or Stimulus–Response Learning

In this second level of complexity, which involves reinforcers, learning may occur. If an organism performs trial-and-error responses and the result of performing one particular response is more likely to obtain a reward, then the response may become linked by a learning process to that stimulus as a result of the reinforcement received. The reward is said to reinforce the response to that stimulus, and we have what is described as stimulus–response or habit learning. The reward acts as a positive reinforcer in that it increases the probability of a response on which it is made contingent. A punisher reduces the probability of a response on which it is made contingent. It should be noted that this is an operational definition, and there is no implication that the punisher feels like anything in particular; the punisher just has, in the learning mechanism, to reduce the probability of responses followed by the punisher.

Stimulus–response or habit learning is typically evident after over-training, and once habits are being executed, the behaviour becomes somewhat independent of the reward value of the goal, as shown in experiments in which the reward is devalued. This is described in more detail in Section 2.1 and Section 4.2.7 *Wanting vs. Liking*. (Stimulus–response habit learning is quite different from action–outcome learning, in which actions are performed and learned to obtain a goal, and the value of the goal therefore influences the actions [6,16,22].)

Once a behaviour becomes automated as a habit, aversive stimuli can be avoided with very little sign of an emotional state.

The dopamine system is implicated in providing the training signal for this type of habit learning implemented in the striatum by encoding reward prediction errors [6,12,49,135]. Evidence that the mesolimbic dopamine system can be more involved in wanting (motivation) than in rewards (liking) is described in Section 7.3.

#### 4.2.3. Rewards and Punishers: Instrumental Goals for Action Towards Which Motivation Is Directed

As soon as we have approach to stimuli at one end of a dimension (e.g., a source of nutrient) and away from stimuli at the other end of the dimension (a lack of nutrients in this case), we can start to wonder when it is appropriate to introduce the terms ‘rewards’ and ‘punishers’ for the stimuli at the different ends of the dimension. By convention, if an animal’s response consists of a fixed response to obtain the stimulus (e.g., locomotion up a chemical gradient), we shall call this a taxis, not a reward. If a fixed behavioural response or action pattern such as skeletomotor freezing and autonomic responses are elicited by a stimulus, they may be adaptive but are essentially stimulus–response reflexes, with no need for an intervening motivational state, such as the representation of a goal to be reached.

On the other hand, if an arbitrary operant action (an instrumental action) can be performed by the animal in order to obtain the stimulus or goal, then we will call this rewarded behaviour, and the stimulus that the animal works to obtain a reward is the goal for the action, and the state of wanting and being willing to work for the goal is a motivational state. The arbitrary operant response can be thought of as any arbitrary action that the animal will perform to obtain the stimulus, the goal. This criterion of an arbitrary operant response is often tested by bidirectionality. For example, if a rat can be trained to either raise its tail or lower its tail in order to obtain a piece of food, then we can be sure that there is no fixed relationship between the stimulus (e.g., the sight of food) and the response or action, as there is in a taxis. I, as well as a number of other authors [136], reserve the term ‘motivated behaviour’ for behaviour in which an arbitrary operant action and an instrumental action will be performed to obtain a reward or to escape from or avoid a punisher; that is, the action is being performed to obtain the goal. This is the action–outcome learning described above in which the anterior cingulate cortex is implicated [8,12]. If this criterion is not met and only a fixed response can be performed, then the term ‘drive’ can be used to describe the state of the animal when it will work to obtain or escape from the stimulus.

We can thus distinguish a first level of approach/avoidance mechanism complexity in a taxis, with a fixed response available for the stimulus; from a second level of complexity in which any arbitrary response (or action) can be performed, in which case we use the term reward when a stimulus is being approached, and punisher when the action is to escape from or avoid the stimulus. The motivational, intervening state is one in which the animal will perform an arbitrary action to obtain a goal. Again, we should distinguish habit-related stimulus–response-implemented instrumental behaviour from action–outcome instrumental behaviour that is under the control of the goal.

The role of natural selection in this process is to guide animals to build sensory systems that will respond to dimensions of stimuli in the natural environment along which actions of the animals can lead to better survival to enable genes to be passed on to the next generation, which is what we mean by fitness. Fitness refers to the fitness of genes, but this must be measured by the effects that the genes have on the organism. The animals must be built by such natural selection to perform actions that will enable them to obtain more rewards, that is to work to obtain stimuli that will increase their (reproductive) fitness. Correspondingly, animals must be built to perform actions that will enable them to escape from or avoid (when learning mechanisms are introduced) stimuli that will reduce their fitness. There are likely to be many dimensions of environmental stimuli along which actions of the animal can alter fitness. Each of these dimensions may be a separate reward–punisher dimension. An example of one of these dimensions might be food reward. It increases fitness to be able to sense nutrient needs, to have sensors that respond to the taste of food, and to perform behavioural responses to obtain such reward stimuli when in that need or motivational state [8,12]. Similarly, another dimension is water reward, in which the taste of water becomes rewarding when there is body-fluid depletion [5].

One aspect of the operation of these reward–punisher systems that these examples illustrate is that with very many reward–punisher dimensions for which actions may be performed, there is a need for a selection mechanism for actions performed to these different dimensions. In this sense, each specific reward and punisher is on a common scale [137] to facilitate the operation of action selection mechanisms. Evolution must set the magnitudes of each of the different reward systems so that each will be chosen for action in such a way as to maximize overall (reproductive) fitness. Food reward must be chosen as the aim for action if some nutrient depletion is present, but water reward as a target for action must be selected if current water depletion poses a greater threat to fitness than the current degree of food depletion. This indicates that for a competitive selection process for rewards, each reward must be carefully calibrated in evolution to have the right value on a common scale for the selection process (but not converted into a common currency) [6]. Other types of behaviours, such as sexual behaviour, must be performed sometimes, but probably less frequently, in order to maximize (reproductive) fitness (as measured by gene transmission into the next generation).

There are many processes that contribute to increasing the chances that a wide set of different environmental rewards will be chosen over a period of time, including not only need-related satiety mechanisms that reduce the rewards within a dimension (such as hunger signals described below) but also sensory-specific satiety mechanisms, which facilitate switching to another reward stimulus (sometimes within and sometimes outside of the same main dimension), and attraction to novel stimuli. Attraction to novel stimuli, i.e., finding novel stimuli rewarding, is one way that organisms are encouraged to explore the multidimensional space within which their genes are operating. The suggestion is that animals should be built to find somewhat novel stimuli rewarding, for this encourages them to explore new parts of the environment in which their genes might perform better than others’ genes. Unless animals are built to find novelty somewhat rewarding, the multidimensional genetic space being explored by genes in the course of evolution might not find the appropriate environment in which they might do better than others’ genes [6]. The primate orbitofrontal cortex contains neurons that respond to novel stimuli [138].

#### 4.2.4. Motivation, Instrumental, Action–Outcome, and Goal-Directed Learning

When behaviour is under the control of the goal, such as the reward or punisher, then we call this motivated behaviour. A test of whether the behaviour is under the control of the goal is reward devaluation. For example, if humans and other animals are fed to satiety with a food, they show sensory-specific satiety for the food, rate its subjective pleasantness as zero, and are no longer motivated to obtain and ingest it. The motivation for other foods not eaten to satiety usually remains. The hallmark of a devaluation experiment showing that a behaviour is under the control of the goal and therefore qualifies for being described as ‘motivated’ is that when the goal is devalued, the human or other animal will not perform an instrumental action to obtain it the first time that the stimulus is presented [8,22] (see Section 2.1 and Section 4.2.7 *Wanting vs. Liking*).

Two stages of learning may be involved in such motivational goal-controlled instrumental learning. Rewards and punishers provide the basis for guiding behaviour within a dimension, and for selecting the dimension towards which action should be directed.

The first stage of learning is stimulus–reinforcer association learning, in which the reinforcing value of a previously neutral, e.g., visual or auditory, stimulus is learned because of its association with a primary reinforcer, such as a sweet or salty taste [139], or a painful touch. This learning is an association between one stimulus, the conditioned or secondary reinforcer, and the primary reinforcer and is thus stimulus–stimulus association learning. This stimulus–reinforcer learning can be very fast, in as little as one trial. For example, if a new visual stimulus is seen and then placed in the mouth and a sweet taste is obtained, an instrumental action such as reaching for the object will be made on the next trial. Moreover, this stimulus–reinforcer association learning can be reversed very rapidly, at least in primates including humans though not in rodents. For example, if subsequently the object is made to taste of salt, then the visual stimulus is no longer reached for, and the stimulus is even likely to be actively pushed away. This stimulus–reinforcer association learning is implemented in the primate including human orbitofrontal cortex and leads to representations of expected value [6,7,12,52].

The second process or stage in this type of learning is instrumental learning of an action (or ‘operant response’) made in order to obtain the reward (or avoid the punisher) indicated by the (discriminative) stimulus. This is action–outcome learning (implemented in brain regions such as the anterior cingulate cortex as described above [6,12,19,44,46]). The outcome could be a primary reinforcer such as the taste of food or the avoidance of an aversive stimulus. Action–outcome learning may be much slower than stimulus–reinforcer learning, for action–outcome learning may involve trial-and-error learning of which action is successful in enabling the individual to obtain the stimulus now associated with reward, or avoid the stimulus now associated with a punisher. However, this second stage may be greatly speeded if an operant response or strategy that has been learned previously to obtain a different type of reward (or avoid a different punisher) can be used to obtain (or avoid) the new stimulus now known to be associated with reinforcement. It is in this flexibility of the action that two-factor learning has a great advantage over stimulus–response learning. The advantage is that any action (even, at its simplest, approach or withdrawal) can be performed once an association has been learned between a stimulus and a primary reinforcer. This flexibility in the action is much more adaptive (and could provide the difference between survival or not) than no learning, as in taxes; or fixed action patterns; or stimulus–response habit learning. The different processes that are involved in instrumental learning are described in more detail by Rolls [6].

Another key advantage of this type of two-stage learning is that after the first stage, the different rewards and punishers available in an environment can be compared in a selection mechanism using the common scale of different rewards and punishers for the comparison and selection process [6,137]. In this type of system, the many dimensions of rewards and punishers (including the reinforcers shown in Table 1) are again the basis on which the selection of an action to perform is made [6].

#### 4.2.5. Gene-Specified Rewards and the Mechanisms of Evolution

Part of the process of evolution can be seen as identifying the factors or dimensions that affect the (reproductive) fitness of an animal and providing the animal with sensors that lead to rewards and punishers that are tuned to the environmental dimensions that influence fitness. The example of sweet or salt taste receptors being set up by evolution to provide reward when physiological nutrient need is present [139] has been given above and shows how genes are involved in specifying motivational states.

We can ask whether there would need to be a separate sensing mechanism tuned to provide primary (unlearned) reinforcers for every dimension of the environment to which it may be important to direct motivational behaviour. (The motivated behaviour has to be directed to climb up the reward gradient to obtain the best reward or to climb a gradient up and away from punishers). It appears that there may not be. For example, in the case of the so-called specific appetites, for perhaps a particular vitamin lacking in the diet, it appears that a type of stimulus–reinforcer association learning may actually be involved, rather than having every possible flavour set up to be a primary reward or punisher. The way that this happens is by a form of association learning. If an animal deficient in one nutrient is fed a food with that nutrient, it turns out that the animal’s physiological state is ‘better’ some time after ingesting the new food, and the animal associates this better physiological state with the taste of that particular food. Later, that food will be chosen. The point here is that the first time the animal is in the deficient state and tastes the new food, that food may not be chosen instead of other foods. It is only after post-ingestive conditioning that, later, that particular food will be selected [140,141,142]. Thus, in addition to a number of specific primary (unlearned) reward systems (e.g., a sweet taste for nutrient needs, a salty taste for salt deficiency [139], and pain for potentially damaging somatosensory stimulation), there may be great opportunity for other arbitrary sensory stimuli to become conditioned rewards or punishers by association with some quite general change in physiological state. The implication here is that a number of bodily signals can influence a general bodily state, and we learn to improve the general state, rather than to treat the signal as a specific reinforcer that directs us to a particular goal. Another example might be social reinforcers. It would be difficult to build in a primary reinforcer system for every possible type of social reinforcer. Instead, there may be a number of rather general primary social reinforcers, such as acceptance within a group, approbation, greeting, face expression, and pleasant touch, which are among the primary rewards; and by association with these primary rewards, other stimuli can become secondary social reinforcers.

To help specify the way in which stimulus–reinforcer association learning operates, a list of what may be in at least some species primary reinforcers is provided in Table 1. The reader will undoubtably be able to add to this list, and it may be that some of the reinforcers in the list are actually secondary reinforcers. The reinforcers are categorized where possible by modality to help the list be systematic. Possible dimensions to which each reinforcer is tuned are suggested.

In Rolls’ theories of motivation and emotion, there may be a set of approximately 100 gene-specified primary reinforcers of the type described in Table 1. Each primary reinforcer accounts for a motivational state in which the reward is the target of an instrumental action, and for the emotional state that is produced when the reward or punisher is or is not received. These motivational and emotional states must all be specific; for example, hunger must increase food reward but not water reward. These reward value systems must be modulated by the correct selective signals; for example, sensors of metabolic state that relate to hunger must increase the reward value of food but not of water. Thus, there must be mechanisms to lead animals, when in a motivational state, to navigate and perform appropriate actions to find a specific reward [143]. The reward is produced by the sensory input produced by taste, smell, flavour, touch, sight, sound, etc., and not by a reduction in the motivational signal. Some of the evidence for this is that very small sensory inputs, such as a drop of food, act as powerful rewards, but reducing hunger by placing food into the stomach produces little reward [6,141]. Consistent with this, reducing the firing of hunger neurons has only a minor rewarding effect [144], so reducing hunger or more generally motivation does not normally act as the reward for instrumental behaviour.

In the reward-based motivational system that I describe, each reward must be scaled to a similar range so that the different rewards are selected at least sometimes by competing in a decision-making process so that each reward can contribute to survival and reproductive success [6]. Motivational behaviour can be seen from this approach as an individual operating with a set of initially gene-specified goals for actions (though subject to learned re-evaluation) which compete in a high-dimensional space of rewards for a decision to be taken about which is most rewarding at the time, depending on modulators such as hunger signals, sensory-specific satiety, etc. [6]. The decision taken will also reflect the costs of the actions required to obtain the different rewards [6]. Evidence about how the underlying mechanisms operate are described in *Emotion and Decision-Making Explained* [6] and elsewhere [7,12].

#### 4.2.6. Biological Economy in the Specification of Rewards and Punishers, for They Can Be Used to Implement Both Motivation and Emotion

We now come to the heart of the adaptive value of my approach to motivation and emotion.

My proposal is that the same gene-specified rewards and punishers can be used for both motivation and emotion. This produces great simplification in the genetic specification of motivation and emotion, for the genes have to specify just one set of primary rewards and punishers. The reward has to be motivating, in that animals need to be built to want to perform actions to obtain rewards. Each gene-specified reward then needs to be modulated by the appropriate motivational state. For example, the motivational state of hunger, which modulates the reward value of the taste, smell, and sight of food, is signalled by many factors including plasma and gut nutrients and metabolic hormones, as described in detail elsewhere [6,7,78,131]. The motivational state of thirst, which modulates the reward value of the taste and sight of water, is signalled by cellular and extracellular fluid volume [5,133,134,145,146]. Factors that influence the reward value of stimuli involved in sexual behaviour are numerous and are typically adaptive for the genes [6,7,147,148]. For example, in males, the reward value of sexual behaviour typically decreases soon after ejaculation, as a further ejaculate in the same female soon would be unlikely to increase markedly the probability of reproductive success, and it may be adaptive to conserve some sperm for a possible opportunity for reproductive success with another female, with sensory-specific satiety here being referred to as the Coolidge effect [6,7,147,148]. The reward value of sexual behaviour in females is also subject to modulation by many factors that influence reproductive success [6,7,147,148]. The key point here is that the value of each type of reward must be modulated by appropriate motivational signals, such as gut and plasma nutrient signals for food reward, cellular and extracellular volume reductions for water reward, and factors such as the probability of reproductive success in passing on genes to the next generation for sex rewards [5,6,7,147,148].

The same set of rewards and punishers when received after, for example, an instrumental action, leads to emotional states, as described above.

The great utility of both emotional and motivational states relating to rewards and punishers is that this is a highly efficient way for behaviour to be organized in that the genes specify stimuli that are rewards and punishers and leave it open to the animal to perform any instrumental action to obtain the reward or avoid the punisher. This is very much more efficient than having genes specify fixed behavioural responses such as fixed action patterns to stimuli, such as pecking at small grains as they may be food. The latter type of mechanism of gene-specified responses can have utility for a few responses to a few stimuli, as in the case of chickens pecking at grains of corn and other fixed action patterns. But the genetic specification of many such stimulus–response pairs would be genetically expensive and would have the great disadvantage that there would be no or little flexibility of the response. Instead, when genes are used to specify rewards and punishers of the type set out in Table 1, then an almost unlimited set of actions can be learned to obtain the rewards or avoid the punishers. For this reason, I argue that the specification of rewards and punishers by genes, rather than fixed behavioural responses such as fixed action patterns, is a major factor in the design of brains for evolutionary success.

These concepts (including that an important way for genes to influence behaviour is by specifying the reward and punishment value of stimuli) were developed and made clear by Rolls [5,6,7,131] but were not featured in *The Selfish Gene* and subsequent books [149,150,151]. These concepts are key to understanding how in practice, genes frequently increase their (selfish) success by specifying stimuli that are rewards and punishers. Operating in this way so that the same genes specify rewards and punishers appropriate for both motivation and emotion and do not specify actions leads to great adaptiveness and elegance in brain design [12,13,131].

#### 4.2.7. Wanting vs. Liking and Goal-Directed Motivational Behaviour

Rolls’ theory of motivation holds that each gene-specified reward is a goal for action, that is, accounts for motivation [131]; and also that when the reward is received, it generates emotion [6,7]. An important attribute of these theories of motivation and emotion is that the same specification of a goal object, a reward, perhaps genetically or by stimulus-reward learning, accounts for both the motivation, which has to be produced if the animal is ever to seek the reward, and the emotion, which is associated with the reward when it is received. This makes for great economy in evolution, for genes are needed to specify goal objects and in so doing have to produce both working to obtain those goal objects (‘wanting’) and the emotional state when the goal object is received or not received (‘liking’) [6].

It is useful in this context to discuss an apparent dissociation between ‘wanting’ and ‘liking’ (or ‘desire’ vs. ‘pleasure’) that has been raised [152,153,154]. ‘Wanting’ or conditioned ‘incentive salience’ effects are used to describe classically conditioned approach behaviour to rewards [153,155], and this learning is implemented via the amygdala and ventral striatum, is under the control of dopamine [16], and contributes to addiction [156]. Conditioned ‘incentive salience’ effects can influence instrumental actions made, for example, to obtain food.

A first point is that Berridge and Robinson [153] suggest that ‘liking’ can be measured by orofacial reflexes such as ingesting sweet solutions or rejecting bitter solutions. There is evidence that brain opioid systems are involved in influencing the palatability of and hedonic reactions to foods in that humans report a reduction in the pleasantness of a sucrose solution following the administration of naltrexone which blocks opiate receptors, but can still discriminate between sucrose solutions [157,158]. One problem here is that orofacial reflexes may reflect brainstem mechanisms that are not at all closely related to the reward value of food, as reflected in instrumental actions performed to obtain food (see Figure 2 and Figure 3). Some of the evidence for this is that these responses occur after decerebration, in which the brainstem is all that remains to control behaviour [159] (with consistent evidence from anencephalic humans [160]). Care must be taken about such inferences as there are many routes to behavioural responses [6,12,161] (Figure 2 and Figure 3).

A second point is that normally, the rated reward value or pleasantness given in humans to food is closely related to instrumental actions performed to obtain food, as shown by the close relation between pleasantness ratings (‘liking’) by humans given a food in a sensory-specific satiety experiment, and whether that food is subsequently eaten in a meal (‘wanting’) [162].

Third, confusion may arise when a stimulus–response habit is formed by overlearning and persists even when the reward is devalued by, for example, feeding to satiety. This persistence of stimulus–response habits after reward devaluation should not necessarily be interpreted as ‘wanting’ when not ‘liking’, for it may just reflect the operation of a stimulus–response habit system that produces responses after overlearning without any guidance from reward, pleasantness, and liking [6,16,161]. Indeed, I emphasize that after overtraining, responses may become inflexibly linked to stimuli, the goals, and the reward value of the goals may no longer be directly influencing behaviour in an ongoing way. If behaviour becomes overlearned and a habit or stimulus–response connection is built up by another brain system (such as the basal ganglia), then animals may make automatic responses that are not goal directed [6,16,161]. There has been considerable confusion in the literature caused by overlooking this point [152,153,163,164,165]. Indeed, just as in the research on the amygdala described above in which LeDoux inferred full emotions from conditioned responses, it is unwarranted and potentially misleading to use subjective emotion-laden words such as ‘wanting’ and ‘liking’ that describe emotional feelings [152,154,155,156,163,164,165] when classically conditioned responses such as Pavlovian-Instrumental Transfer, orofacial reflexes, and stimulus–response habits are what has been measured [6,16,161]. The fact that behaviour can become stimulus-response and is therefore no longer under the control of the goal need not surprise us. Normally, and certainly during learning before habits set in, we want a goal, and when we obtain the goal we like it: goal stimuli normally specify what is wanted and what is liked. Indeed, my theory is that normally, we want because we like. This is inherent in my theory, for the genes that make a stimulus (such as a sweet taste) rewarding (i.e., wanted, a goal for action) also make the stimulus liked (i.e., accepted, with a subjective correlate of pleasure, pleasantness, and affective liking).

My approach is that I propose that liking, defined by pleasantness ratings of stimuli, is normally very closely related to wanting, that is being willing to perform a behaviour (instrumental actions) to obtain a reward of the pleasant stimulus [6,131]. Thus, motivational behaviour is normally controlled by reward stimuli or goals (unless the behaviour is overlearned), and motivational state (e.g., hunger) modulates the reward value of unconditioned and conditioned stimuli such as the taste and sight of food. Thus, normally, liking a goal object and wanting it are different aspects of how reward systems control instrumental behaviour, and this follows from the approach to a gene-specified goal or value representations which, in a unifying way, account for wanting a goal, and liking the goal object when it is obtained [6,7,12,131]. For further clarification, consider a probabilistic decision-making task in which the probability *p* of obtaining a reward outcome (such as the taste of food) is 0.5, and the reward outcome value is 1 if the reward is delivered (e.g., fruit juice), and it is 0 when the reward is not delivered. Then, the expected (reward) value when the offer is made is 0.5 (expected reward value = *p* × outcome value [6]), and the value of the motivational state at that time (which is *before* the outcome is known) is 0.5. Then, later in the trial, the affective/emotional state (which is *after* the outcome is delivered) is one for the fruit juice reward, and zero if nothing is obtained as the outcome.

Thus, it is possible to identify the brain mechanisms involved in ‘wanting’ and ‘liking’ experimentally and to distinguish them from the classically conditioned ‘incentive salience’ stimuli that influence approach, instrumental actions, and ‘appetitive’ behaviour, which are implemented in part separately from the reward systems that are activated by a primary reinforcer such as the taste of food during ‘consummatory’ behaviour [6,16]. Incentive salience effects are considered further in Section 7.3.

## 5. Some Implications and Extensions of the Understanding of Emotion, Motivation, and Their Brain Mechanisms

### 5.1. Top-Down Cognitive Effects on Reward Value and Affective Responses, for Example, on the Reward Value and Pleasantness of Taste, Olfactory, and Flavour Stimuli

To what extent does cognition influence the reward value of stimuli, and how far down into the sensory system does the cognitive influence reach? Alternatively, is the reward value in brain regions such as the orbitofrontal cortex independent of cognitive factors, with reward value being interfaced to cognition in other, perhaps language-related, brain regions? What are the relations between cognition and emotion?

We discovered that word-level cognitive effects have top-down modulatory effects on reward value processing in the orbitofrontal cortex and anterior cingulate cortex. This was shown for olfactory [166], taste [91], and touch and the sight of touch [100] reward value. For example, a standard test odour (isovaleric acid combined with cheddar cheese odour, presented orthonasally using an olfactometer) was paired with a descriptor word on a screen, which on different trials was “Cheddar cheese” or “Body odor”. Participants rated the affective value of the standard test odour and isovaleric acid, as significantly more pleasant when labelled “Cheddar Cheese” than when labelled “Body odor”, and these effects reflected activations in the medial orbitofrontal cortex and the pregenual anterior cingulate cortex [166]. The implication is that cognitive factors can have profound effects on our responses to the reward value and subjective reported pleasantness of olfactory stimuli in that these effects are manifest quite far down into reward value processing (in the orbitofrontal cortex) so that hedonic representations of odours are affected [166].

Similar cognitive effects and mechanisms have also been found for the taste and flavour of food, where the cognitive word-level descriptor was, for example, ‘rich delicious flavor’, and activations to flavour were increased in the orbitofrontal cortex and regions to which it projects including the pregenual anterior cingulate cortex and ventral striatum, but were not influenced in the insular primary taste cortex where activations reflected the rated intensity (related to the concentration) of the stimuli [91].

For the sight of touch, the cognitive modulation was produced by word labels, ‘Rich moisturizing cream’ or ‘Basic cream’, while cream was being applied to the forearm, or was seen being applied to a forearm. The cognitive labels influenced the activations to the sight of touch and also the correlations with pleasantness in the pregenual anterior cingulate/orbitofrontal cortex and ventral striatum [100].

The wider implication of these discoveries is that our cognitive processes can actually modulate the representation of reward value and subjective pleasantness in brain regions involved in reward value representations such as the orbitofrontal and pregenual anterior cingulate cortices, and this can potentially provide important ways in which the appreciation of other rewards such as music, art, and esthetics can be influenced by cognitive factors acting on the reward value representations in parts of the brain that represent reward value and subjective pleasantness. In this way, the appropriate top-down cognitive bias could enhance the pleasure being experienced.

The mechanisms of top-down cognitive modulation are understood as biassed activation being applied to the orbitofrontal cortex from brain regions such as the prefrontal cortex that maintain the biassing information in short-term memory [12,13,167,168,169,170,171,172,173,174].

### 5.2. Effects of Top-Down Selective Attention to Affective Value Versus Intensity on Representations of Stimuli Including Those Involved in Taste, Olfactory, and Flavour Processing

We have found that in humans with taste, flavour, and olfactory food-related stimuli, selective attention to pleasantness modulates representations in the orbitofrontal cortex, whereas selective attention to intensity modulates activations in areas such as the primary taste cortex [62,92,174,175,176,177].

This differential biassing of brain regions engaged in processing a sensory stimulus depending on whether the cognitive or attentional demand is for affect-related vs. more sensory-related processing may be an important aspect of cognition and attention which has implications for how strongly the reward system is driven by food and thus for eating and the control of appetite, but also for other types of reward [6,44,92,175,178].

The wider implication is that top-down attention directed to the reward value and subjective pleasantness of stimuli can enhance activations to these stimuli in reward-related brain regions, and this has potential applications to enhance the subjective pleasantness of many types of reward, including esthetic types of rewards (e.g., music and art). Attention applied in this way may divert brain systems from maintaining unpleasant ruminating events in memory, and this is of potential use in, for example, the treatment of depression and other unpleasant states including pain [7,10,12,179].

The mechanisms of top-down attentional modulation are understood as biassed competition and biassed activation being applied to the orbitofrontal cortex from brain regions such as the prefrontal cortex that maintain the biassing information in short-term memory [12,13,62,167,168,169,170,171,172,173,174].

### 5.3. Individual Differences in the Reward Systems, Evolution, and Personality

An important hypothesis is that different humans may have reward systems that differ in how strongly each of their reward systems are activated, driven by the sensory and cognitive factors that make stimuli attractive. In a test of this, we showed that activations to the sight and flavour of chocolate in the orbitofrontal and pregenual cingulate cortexes were much higher in chocolate cravers than non-cravers [180], though there were no differences at the level of the insular taste cortex where taste perception is represented. This provides evidence that differences in specific reward systems and not necessarily in earlier sensory processing can lead to individual differences in behaviour to different types of rewarding stimuli [6,7]. This concept that individual differences in responsiveness to food reward are reflected in brain activations in regions related to the control of food intake [180,181] may provide a way for understanding and helping to control food intake and obesity [6,7,78,178].

But the concept is much more general than this. The wider implication is that part of the way in which evolution operates is by utilizing natural variation in each of the specific reward systems (examples of which are shown in Table 1) and selecting for reward systems with sensitivities that lead to reproductive success. This results in each individual having a different set of sensitivities of perhaps 100 different gene-specified reward systems of the type shown in Table 1 [6,7]. This approach to understanding many aspects of personality in terms of differential sensitivity to different rewards and punishers and the omission and termination of different rewards and punishers arising in evolution is developed elsewhere [8].

### 5.4. A Reasoning, Rational, Route to Action

Routes to action that are related to the emotional system are described in Section 3 and are indicated in Figure 2 and Figure 3.

A main route to action in humans, and perhaps some other species, involves a computation with many “if … then” statements to implement a multi-step plan to obtain a reward. In this case, the reward may actually be *deferred* as part of the plan, which might involve working first to obtain one reward, and only then to work for a second more highly valued reward, if this was thought to be an overall optimal strategy in terms of resource usage (e.g., time and effort). In this case, syntax is required, because the many symbols (e.g., names of people) that are part of the plan must be correctly linked or bound. Such linking might be of the following form: “if A does this, then B is likely to do this, and this will cause C to do this …”. The requirement of syntax for this type of planning implies that the involvement of language systems in the brain is required for this type of planning [6,7,12]. Thus, the explicit language system in humans may allow working for deferred rewards by enabling the use of a one-off, individual, plan appropriate for each situation.

Another building block for such planning operations in the brain may be the type of short-term memory in which the prefrontal cortex is involved. This short-term memory may be for example in non-human primates of where in space a response has just been made. A development of this type of short-term response memory system in humans to enable multiple short-term memories to be held in place correctly, preferably with the temporal order of the different items in the short-term memory coded correctly, may be another building block for the multiple step “if … then” type of computation in order to form a multiple step plan. Such short-term memories are implemented in the (dorsolateral and inferior convexity) prefrontal cortex of non-human primates and humans [12,13,182,183,184,185,186,187] and may be part of the reason why prefrontal cortex damage impairs planning [188].

#### 5.4.1. Decisions Between the Emotional and Reasoning Systems

The question then arises of how decisions are made in animals such as humans that have both the reward-based emotional systems and the explicit, rational, planning systems [6,7,12,189,190,191]. One particular situation in which the first, partly implicit, system may be especially important is when rapid reactions to stimuli with reward or punishment value must be made, for the direct connections from structures such as the orbitofrontal cortex to the basal ganglia may allow for rapid actions [6]. Another is when there may be too many factors to be taken into account easily by the explicit, rational planning system, when the implicit system may be used to guide action. In contrast, when the implicit system continually makes errors, it would then be beneficial for the organism to switch from action based on obtaining what the orbitofrontal cortex system decodes as being the most positively rewarding choice currently available, to the explicit conscious control system that can evaluate with its long-term planning algorithms what action should be performed next. Indeed, it would be adaptive for the explicit system to regularly assess performance using the goal-based and habit-based instrumental systems and to switch itself to control behaviour quite frequently, as otherwise, the adaptive value of having the explicit system would be less than optimal.

There may also be a flow of influence from the explicit, verbal system to the implicit system, in that the explicit system may decide on a plan of action or strategy and exert an influence on the implicit system that will alter the reinforcement evaluations made by and the signals produced by the implicit system using, for example, top-down cognitive control [6,7].

It may be expected that there is often a conflict between these systems in that the first, implicit emotional system is able to guide behaviour particularly to obtain the greatest immediate reinforcement, whereas the explicit system can potentially enable immediate rewards to be deferred and longer-term, multi-step plans to be formed [190,191]. This type of conflict will occur in animals with a syntactic planning ability, that is in humans and any other animals that have the ability to process a series of “if … then” stages of planning. This is a property of the human language system, and the extent to which it is a property of non-human primates is not yet fully clear. In any case, such conflict may be an important aspect of the operation of at least the human mind, because it is so essential for humans to correctly decide, at every moment, whether to invest in a relationship or a group that may offer long-term benefits, or whether to directly pursue immediate benefits [6,7,190,191].

Thus, the thrust of the argument [6,7,8,12,39,191] is that that much complex animal including human behaviour can take place using the implicit, emotional, often unconscious, route to action. We should be very careful not to postulate intentional states (i.e., states with intentions, beliefs, and desires) unless the evidence for them is strong, and it seems to me that a flexible, one-off linguistic processing system that can handle propositions is needed for intentional states. What the explicit linguistic system does allow is exactly this flexible, one-off, multi-step planning ahead type of computation, which allows us to defer immediate rewards based on such a plan.

#### 5.4.2. The Selfish Gene vs. the Selfish Phenotype and Evolution

I have provided evidence above that there are two main routes to decision-making and goal-directed action. The first route selects actions by gene-defined goals for action and is closely associated with emotion. The second route involves multi-step planning and reasoning, which requires syntactic processing to keep the symbols involved at each step separate from the symbols in different steps [12,39,190,191]. (This second route is used by humans and perhaps by closely related animals.) Now the ‘interests’ of the first and second routes to decision-making and action are different. As argued very convincingly by Richard Dawkins in *The Selfish Gene* [192] and by others [193,194,195], many behaviours occur in the interests of the survival of the genes not of the individual (nor of the group), and much behaviour can be understood in this way.

I have extended this approach by arguing that an important role for some genes in evolution is to define the goals (i.e., the rewards and punishers) for actions that will lead to the better survival of those genes; that emotions are the states associated with these gene-defined goals; and that the defining of goals for actions rather that actions themselves is an efficient way for genes to operate, as it leaves the flexibility of choice of action open while the animal is alive [6,7]. This provides a great simplification of the genotype as action details do not need to be specified, just rewarding and punishing stimuli, and also the flexibility of action in the face of changing environments faced by the genes. This is a useful and interesting advance beyond what was considered in *The Selfish Gene* and later books [149,150,151,192]. In any case, the interests that are implied when the first route to action is chosen are those of the “selfish genes”, not those of the individual, the phenotype.

However, the second route to action allows, by reasoning, decisions to be taken that might not be in the interests of the genes, might be longer-term decisions, and might be in the interests of the individual, the phenotype [6,12,189]. An example might be a choice not to have children but instead to devote oneself to science, medicine, music, or literature. The reasoning and the rational system presumably evolved because taking longer-term decisions involving planning rather than choosing a gene-defined goal might be advantageous at least sometimes for genes. But an unforeseen consequence of the evolution of the rational system might be that the decisions would, sometimes, not be to the advantage of any genes in the organism. After all, evolution by natural selection operates by utilizing genetic variations like a blind watchmaker [149]. In this sense, the interests when the second route to decision-making is used are at least sometimes those of the “selfish phenotype”. Hence, the decision-making referred to above is between a first system where the goals are gene defined, and a second rational system in which the decisions may be made in the interests of the genes or in the interests of the phenotype and not in the interests of the genes.

Now what keeps the decision-making between the “Selfish Genes” and the “Selfish Phenotype” more or less under control and in balance? If the second rational system chose too often for the interests of the “Selfish Phenotype”, the genes in that phenotype would not survive over generations. Having these two systems in the same individual will only be stable if their potency is approximately equal so that sometimes decisions are made with the first route and sometimes with the second route. If the two types of decision-making, then, compete with approximately equal potency, and sometimes one is chosen and sometimes the other, then this is exactly the scenario in which stochastic processes in the decision-making mechanism are likely to play an important role in the decision that is taken [6,8,12,13,167,190,191]. The same decision, even with the same evidence, may not be taken each time a decision is made because of noise in the system.

The system itself may have some properties that help to keep the system operating well. One is that if the second rational system tends to dominate the decision-making too much, the first gene-based emotional system might fight back over generations of selection and enhance the magnitude of the reward value specified by the genes so that emotions might actually become stronger as a consequence of them having to compete in the interests of the selfish genes with the rational decision-making processes.

Another property of the system may be that sometimes the rational system cannot gain all the evidence that would be needed to make a rational choice. Under these circumstances the rational system might fail to make a clear decision, and under these circumstances, basing a decision on the gene-specified emotions is an alternative. Indeed, Damasio [130] argued that under circumstances such as this, emotions might take an important role in decision-making. In this respect, I agree with him, basing my reasons on the arguments above. He called emotional feelings gut feelings and, in contrast to me, hypothesized that actual feedback from the gut was involved. His argument seemed to be that if the decision was too complicated for the rational system, then rely on outputs sent to the viscera, and whatever is sensed by what they send back could be used in the decision-making process and would account for the conscious feelings of the emotional states. My reading of the evidence is that the feedback from the periphery is not necessary for emotional decision-making or for the feelings, nor would it be computationally efficient to put the viscera in the loop given that the information starts from the brain, but that is a matter considered in Section 6.1 and elsewhere [6,7,196].

Another property of the system is that the interests of the second rational system, although involving a different form of computation, should not be too far from those of the gene-defined emotional system for the arrangement to be stable in evolution by natural selection. One way that this could be facilitated would be if the gene-based goals felt pleasant or unpleasant in the rational system and in this way contributed to the operation of the second rational system. This is something that I propose is the case [6,7,8,39,189].

#### 5.4.3. A Higher-Order Syntactic Thought (HOST) Approach to Consciousness Including Emotional Feelings

If the multi-step syntactic reasoning/planning system fails, there may be a credit assignment problem in that the faulty step in the series of steps needs to be identified. It has been argued that this computation requires a higher-order thought system that can think about the first-order thought (the plan) and can correct it. This higher order thought system needs syntax, as it has to perform computations on the first-order syntactic thoughts, such as the plan. I have proposed that it is a property of this Higher-Order Syntactic Thought system that when it operates, it would feel like something to be thinking about one’s own first-order thoughts, and that is the basis for my HOST theory of consciousness [39,63,189,197]. It is suggested that anything that is being dealt with by the HOST computational system becomes conscious, and that is also how qualia, raw sensory feels, and emotional feelings are produced [39].

Consistent with this approach [39], there is effective connectivity from the human lateral orbitofrontal cortex to language areas implicated in syntactic processing including inferior frontal gyrus regions in and close to Broca’s area [12,20,61,67] (Figure 4). Further relevant evidence is that the orbitofrontal cortex but much less the amygdala has backprojections to neocortical regions [61], and that orbitofrontal cortex damage in humans impairs subjectively experienced emotion [102,103], whereas the human amygdala is not strongly implicated in subjectively experienced emotion [35,38,125]. Consistent also with this approach is the approximately linear relation between the fMRI BOLD signal in orbitofrontal cortex regions and the subjective, conscious ratings of the pleasantness of stimuli [6,12,91,137,175,198,199,200]. A possible implication of this and what is shown in Figure 2, Figure 3 and Figure 4 might be that a pathway from the orbitofrontal cortex to cortical systems involved in higher-order syntactic thought processing, which implies a computation but is not necessarily human language-based, is involved in experienced emotional feelings. As is made clear, this is a hypothesis only and should not be taken to have practical implications [39]. One possible way forward that I propose is to analyze fully the brain systems involved in subjective emotional feelings in humans, which can be reported, and then assess to what extent the key computational brain systems involved in emotional feelings in humans can be identified and operate similarly in non-humans. Further discussion of these issues is provided elsewhere [201,202].

This raises the issue of what the relation is between the mind and the brain [203,204,205,206]. In the neuroscience-based approach that I propose for the relation between the mind and the brain, the proposal is that events at the sub-neuronal, neuronal, and neuronal network levels take place simultaneously to perform a computation that can be described at a high level as a mental state with content about the world [207,208]. It is argued that as the neural and computational processes at the different levels of explanation take place at the same time, they are linked by a non-causal relationship: causality can best be described in brains as operating within levels across time, but not between levels. This mind–brain theory allows mental events to be different in kind from the mechanistic events that underlie them but does not lead one to argue that mental events cause brain events, or vice versa; there are different levels of explanation of the operation of the computational system [207,208]. This computational neuroscience levels of explanation approach to causality [12] provides an opportunity to proceed beyond Cartesian dualism [203] and physical reductionism [206] in considering the relations between the mind and the brain [39,207,208].

### 5.5. The Neurology of Human Emotion: The Orbitofrontal Cortex Compared to the Amygdala

Differences between the human orbitofrontal cortex and the amygdala and how their different output systems account for them having different roles in emotion are considered elsewhere [8,12,61]. One difference is that the human orbitofrontal cortex has more connectivity back to cortical regions including cortical regions for language than the amygdala [61]. Moreover, although the macaque is overall a good model of the system-level organization of the human brain, the rodent brain is much less so, as described in section 19.10 of *Brain Computations and Connectivity* [12].

### 5.6. A Psychiatric Disorder of Emotion: Depression

The orbitofrontal cortex is described here as having important roles in emotion in terms of functions it performs in rapid and reversible stimulus-reward learning and in providing routes to explicit declarative systems. Consistent with this, it is proposed that the orbitofrontal cortex is important in a major disorder of human emotion, depression, with the approach and evidence described elsewhere [7,9,10,11,51,95,179,209,210,211,212].

An important concept in relation to understanding emotion and its disorders is that maintaining language-related ruminating thoughts because of cortico-cortical feedback loops involving attractor networks between the orbitofrontal cortex and language-related systems may make depressive states particularly severe in humans [7,179].

### 5.7. Role of Reward and Emotion in Episodic and Semantic Memory and in Memory Consolidation

The human orbitofrontal cortex connecting with the vmPFC and anterior cingulate cortex provides a route to the hippocampus for reward and emotional value to be incorporated into episodic memory, enabling memory of where a reward was seen [20,64,213] (Figure 4). In particular, Figure 4 shows how reward regions of the orbitofrontal cortex, vmPFC (pOFC, 10r, 10v), and pregenual anterior cingulate cortex (a24 and p32), as well as the punishment/non-reward regions of the lateral orbitofrontal cortex (47m), have effective connectivity with the hippocampus, entorhinal cortex, and perirhinal cortex. Consistent with this, some neurons in the primate hippocampus respond to a combination of a spatial view and the reward value that is available at that location in a scene location—reward memory task [214]. It is argued that reward, punishment, and more generally emotional value are important components of episodic memory [64]. Even more than this, it is argued that emotional value may influence the consolidation of memories through the connectivity of the orbitofrontal cortex with the basal forebrain cholinergic neurons [12,20,64]. This may be another important and adaptive function of emotions.

## 6. Comparison with Other Theories of Emotion

For completeness, I now outline some other theories of emotion and compare them with the above (Rolls’) theory of emotion. Surveys of some of the approaches to emotion that have been taken in the past are provided by Strongman [215] and Keltner, Oatley and Jenkins [216].

### 6.1. The James–Lange and Other Bodily Theories of Emotion Including Damasio’s Theory

James [217] believed that emotional experiences were produced by sensing bodily changes, such as changes in heart rate or in skeletal muscles. Lange [218] had a similar view, although he emphasized the role of autonomic feedback (for example from the heart) in producing the experience of emotion. The theory, which became known as the James–Lange theory, suggested that there are three steps in producing emotional feelings. The first step is elicitation by the emotion-provoking stimulus of peripheral changes, such as skeleto-muscular activity to produce running away and autonomic changes, such as alteration of heart rate. But, as pointed out above, the theory leaves unanswered perhaps the most important issue in any theory of emotion: Why do some events make us run away (and then feel emotional), whereas others do not? This is a major weakness of this type of theory. The second step is the sensing of the peripheral responses (e.g., running away and altered heart rate). The third step is the elicitation of the emotional feeling in response to the sensed feedback from the periphery.

The history of research into peripheral theories of emotion starts with the fatal flaw that step one (the question of which stimuli elicit emotion-related responses in the first place) leaves unanswered this most important question. The history continues with the accumulation of empirical evidence that has gradually weakened more and more the hypothesis that peripheral responses made during emotional behaviour have anything to do with producing the emotional behaviour (which has largely already been produced anyway according to the James–Lange theory) or the emotional feeling. Some of the landmarks in this history are described by Rolls [6].

First, the peripheral changes produced during emotion are not sufficiently distinct to be able to carry the information that would enable one to have subtly different emotional feelings to the vast range of different stimuli that can produce different emotions. The evidence suggests that by measuring many peripheral changes in emotion, such as heart rate, skin conductance, breathing rate, and hormones such as adrenaline and noradrenaline (known in the United States by their Greek names epinephrine and norepinephrine), it may be possible to make coarse distinctions between, for example, anger and fear but not much finer distinctions [216]. Brain processing must of course produce the somewhat different autonomic responses in the first place, and there is evidence that the orbitofrontal and anterior cingulate cortices, perhaps acting via an insular visceral cortex region, are involved in producing autonomic responses [53,219]. Of course there are pathways from the viscera to the brain, and visceral changes can influence the brain [53,54,219], but whether those visceral changes are in the normal causal chain for the elicitation of emotional states is much more difficult to prove.

Second, when emotions are evoked by imagery, then the peripheral responses are much less marked and distinctive than during emotions produced by external stimuli [220,221]. This makes sense in that although an emotion evoked by imagery may be strong, there is no need to produce strong peripheral responses, because no behavioural responses are required.

Third, the disruption of peripheral responses and feedback from them either surgically (for example in dogs [222,223] or as a result of spinal cord injury in humans [224,225]) does not abolish emotional responses. What was found was that in some patients, there was apparently some reduction in emotions in some situations [224], but this could be related to the fact that some of the patients were severely disabled (which could have produced its own consequences for emotionality), and in many cases, the patients were considerably older than before the spinal cord damage, and this could have been a factor. What was common to both studies was that emotions could be felt by all the patients; and in some cases, emotions resulting from mental events were even reported as being stronger [224,225].

Fourth, when autonomic changes are elicited by injections of, for example, adrenaline or noradrenaline, particular emotions are not produced. Instead, the emotion that is produced depends on the cognitive decoding of the reinforcers present in the situation, such as an actor who insults your parents to make you angry, or an actor who plays a game of hula hoop to make you feel happy [226]. In this situation, the hormone adrenaline or noradrenaline can alter the magnitude of the emotion, but not which emotion is felt. This is further evidence that it is the decoded reinforcement value of the input stimulus or events that determines which emotion is felt. The fact that the hormone injections produced some change in the magnitude of an emotion is not very surprising. If you felt your heart pounding for no explicable reason, you might wonder what was happening and therefore react more or abnormally.

Fifth, if the peripheral changes associated with emotion are blocked with drugs, then this does not block the perception of emotion [227].

Sixth, it is found that in normal life, behavioural expressions of emotion (for example smiling when at a bowling alley) do not usually occur when one might be expected to feel happy because of a success, but instead occur when one is looking at one’s friends [228]. These body responses, which can be very brief, thus often serve the needs of communication or action, not of producing emotional feelings.

Despite this rather overwhelming evidence against an important role for body responses in producing emotions or emotional feelings, Damasio [130] effectively tried to resurrect a weakened version of the James–Lange theory of emotion from the 19th century by arguing with his *somatic marker hypothesis* that after reinforcers have been evaluated, a bodily response (‘somatic marker’) normally occurs, then this leads to a bodily feeling, which in turn is appreciated by the organism to then make a contribution to the decision-making process. [In the James–Lange theory, it was emotional feelings that depend on peripheral feedback; for Damasio, it is the decision of which behavioural response to make that is normally influenced by the peripheral feedback. A quotation from Damasio (1994, p. 190) [130] is as follows: “The squirrel did not really think about his various options and calculate the costs and benefits of each. He saw the cat, was jolted by the body state, and ran.” Here, it is clear that the pathway to action uses the body state as part of the route. Damasio would also like decisions to be implemented using the peripheral changes elicited by emotional stimuli. Given all the different reinforcers that may influence behaviour, Damasio [130] even suggests that the net result of them all is reflected in the net peripheral outcome, and the brain can sense this net peripheral result and thus know what decision to take.] The James–Lange theory has a number of major weaknesses just outlined that apply also to the somatic marker hypothesis.

The somatic marker hypothesis postulates that emotional decision-making is facilitated by peripheral feedback from, for example, the autonomic nervous system. In a direct test of this, emotional decision-making was measured using the Iowa Gambling Task [109] in patients with pure autonomic failure [229]. In this condition, there is the degeneration of the peripheral autonomic system, and thus autonomic responses are severely impaired, and there can be no resulting feedback to the brain. It was found that performance in the Iowa Gambling Task was not impaired, nor were many other tests of emotion and emotional performance, including face expression identification, theory of mind tasks of social situations, and social cognition tasks. Thus, emotional decision-making does not depend on the ongoing feedback from somatic markers related to autonomic function. Damasio might argue that feedback from the autonomic system is not actually important, and it is feedback from skeletomotor responses such as arm movements or muscle tension that is important. He might also argue that autonomic feedback is not usually necessary for emotional decision-making, because it can be ‘simulated’ by the rest of the brain. However, the study by Heims et al. [229] does show that ongoing autonomic feedback is not necessary for normal emotional decision-making, and this leaves the somatic marker hypothesis more precarious.

Part of the evidence for the somatic marker hypothesis was that normal participants in the Iowa Gambling Task were described as deciding advantageously before knowing the advantageous strategy [110]. The interpretation was that they had implicit (unconscious) knowledge implemented via a somatic marker process that was used in the task, which was not being solved by explicit (conscious) knowledge. Maia and McClelland [196,230] however showed that with more sensitive questioning, normal participants at least had available to them explicit knowledge about the outcomes of the different decks that was as good as or better than the choices made, weakening the arguments that the task was being solved implicitly and using somatic markers [109,110].

Another argument against the somatic marker hypothesis is that there can be dissociations between autonomic and other indices of emotion, thus providing evidence that behaviour may not follow from autonomic and other effects. For example, lesions of different parts of the amygdala influence autonomic responses and instrumental behaviour differently [6,7].

Another major weakness, which applies to both the James–Lange theory and to Damasio’s somatic marker hypothesis, and to the roles of feedback from the autonomic system to the brain [54], is that they do not take account of the fact that once an information processor has determined that a response should be made or inhibited based on reinforcement association, a function attributed in Rolls’ theory of emotion [6,7] to the orbitofrontal cortex, it would be very inefficient and noisy to place in the execution route a peripheral response, and transducers to attempt to measure that peripheral response, itself a notoriously difficult procedure. Even for the cases when Damasio [130] might argue that the peripheral somatic marker and its feedback can be bypassed using conditioning of a representation in, e.g., the somatosensory cortex to a command signal (which might originate in the orbitofrontal cortex), he apparently would still wish to argue that the activity in the somatosensory cortex is important for the emotion to be appreciated or to influence behaviour. (Without this, the somatic marker hypothesis would vanish.) The prediction would apparently be that if an emotional response was produced to a visual stimulus, then this would necessarily involve activity in the somatosensory cortex or other brain region in which the ‘somatic marker’ would be represented. This prediction could be tested (for example in patients with somatosensory cortex damage), but it seems most unlikely that an emotion produced by a visual reinforcer would require activity in the somatosensory cortex to feel emotional or to elicit emotional decisions. However, it has been found that the more damage there is to somatosensory cortex, the greater the impairment in the emotional state reported by patients [231]. However, the parts of the somatosensory system that appear to be damaged most frequently in the patients with emotional change are often in the anterior and ventral extensions of the somatosensory cortex in the insular and nearby areas, and it would be useful to know whether this damage interrupted some of the connections or functions of the orbitofrontal cortex areas just anterior to it.

More recently, Damasio has stated the somatic marker hypothesis in a weak form, suggesting that somatic markers do not even reflect the valence of the reinforcer, but just provide a signal that depends on the intensity of the emotion, independently of the type of emotion. On this view, the role of somatic markers in decision-making would be very general, providing, as Damasio says, just a jolt to spur the system on (A.R. Damasio, paper delivered at the 6th Annual Wisconsin Symposium on Emotion, April 2000).

The alternative view proposed here and elsewhere [2,6,7,8,232,233] is that where the reinforcement value of the visual stimulus is decoded, namely in the orbitofrontal cortex and the amygdala, is the appropriate part of the brain for outputs to influence behaviour (via, e.g., the orbitofrontal to cingulate cortex and orbitofrontal-to-striatal connections), and the orbitofrontal cortex, amygdala, and brain structures that receive connections from them are the likely places where neuronal activity is directly related to emotional states.

### 6.2. Appraisal Theory

Appraisal theory [216,234,235] generally holds that two types of appraisal are involved in emotion. Primary appraisal holds that “an emotion is usually caused by a person consciously or unconsciously evaluating an event as relevant to a concern (a goal) that is important; the emotion is felt as positive when a concern is advanced and negative when a concern is impeded” [236]. The concept of appraisal presumably involves the assessment of whether something is a reward or punisher, that is, whether it will be worked for or avoided. The description in terms of rewards and punishers adopted here (and by Rolls [70]) simply seems much more precisely and operationally specified. If primary appraisal is defined with respect to goals, it might be helpful to note that goals may just be the reinforcers specified in Rolls’ theory of emotion [1,2,5,70], and if so, the reinforcer/punisher approach provides clear definitions of goals. Secondary appraisal is concerned with coping potential, that is, whether, for example, a plan can be constructed, and how successful it is likely to be.

Scherer [237] summarizes his appraisal theory approach as follows. He suggests that there are four major appraisal objectives to adaptively react to a salient event:

(a) Relevance: How relevant is this event for me? Does it directly affect me or my social reference group?
(b) Implications: What are the implications or consequences of this event, and how do they affect my well-being and my immediate or long-term goals?
(c) Coping Potential: How well can I cope with or adjust to these consequences?
(d) Normative Significance: What is the significance of this event for my self-concept and for social norms and values?

To attain these objectives, the organism evaluates the event and its consequences on a number of criteria or stimulus evaluation checks, with the results reflecting the organism’s subjective assessment (which may well be unrealistic or biassed) of consequences and implications on a background of personal needs, goals, and values. Scherer [237] states that an important feature of the model is that it does not include overt instrumental behaviour. Instead, he sees emotion as a reaction to significant events that prepare action readiness and different types of alternative, possibly conflicting action tendencies but not as a sufficient cause for their execution. This is a clear difference from my theory in that in my theory emotions are states that have a key role in brain design by providing a way for stimuli to produce states that are the goals for instrumental actions. Of course, stimuli that are instrumental reinforcers, goals for action, can also produce adaptive autonomic and skeletomotor reflexes (such as freezing), but these are responses and can be classically conditioned, but they do not require intervening goal-related representations or states, which are emotional and motivational states.

I note that appraisal theory is in many ways quite close to the theory that I outline here and elsewhere [1,2,5,70], and I do not see them as rivals. Instead, I hope that those who have an appraisal theory of emotion will consider whether much of what is encompassed by primary appraisal is not actually rather close to assessing whether stimuli or events are instrumental reinforcers and whether much of what is encompassed by secondary appraisal is rather close to taking into account the actions that are possible in particular circumstances.

An aspect of some flavours of appraisal theory with which I do not agree is that emotions as one of their functions release particular actions, which seem to make a link with species-specific action tendencies or responses, or ‘fixed action patterns’ [238,239] or more ‘open motor programs’ [240]. I argue that rarely are behavioural responses programmed by genes, but instead, genes optimize their effects on behaviour if they specify the goals for (flexible) actions, that is, if they specify rewards and punishers [6]. The difference is quite considerable in that specifying goals is much more economical in terms of the information that must be encoded in the genome; and specifying goals for actions allows much more flexibility in the actual actions that are produced. Of course I acknowledge that there is some preparedness to learn associations between particular types of secondary and primary reinforcers [241], and I see this just as an economy of sensory–sensory convergence in the brain, whereby, for example, it does not convey much advantage to be able to learn that flashing lights (as contrasted with the taste of a food just eaten) are followed by sickness.

### 6.3. Panksepp’s Theory of Emotion

Panksepp’s approach to emotion had its origins in neuroethological investigations of brainstem systems that when activated lead to behaviours like fixed action patterns, including escape, flight, and fear behaviours [238,239]. Using evidence from brain stimulation that elicits behaviours, he has postulated that there are a set of basic emotions, including, for example, seeking, rage, fear, lust, care, panic/grief, and play. He argued that these are ‘natural kinds’, things that exist in nature as opposed to being inventions (constructions) of the human mind. My view is that there are not a few basic emotions, and emotions do not involve fixed action patterns as these do not require intervening emotional states to support goal-directed instrumental actions, and that emotions can be classified based on the specific reinforcer, the specific reinforcement contingency, the actions that are available, etc., as described in Rolls’ theory of emotion [6,7].

### 6.4. Dimensional, Categorical, and Other Theories of Emotion

Dimensional and categorical theories of emotion suggest that there are a number of fundamental or basic emotions. Charles Darwin, for example, in his book *The Expression of the Emotions in Man and Animals* [242] showed that some basic expressions of emotion are similar in animals and humans. Some of the examples he gave are shown in Table 1. His focus was on the continuity between animals and humans of how emotion is expressed.

In a development of this approach, Ekman [240,243] has suggested that humans categorize face expressions into a number of basic categories that are similar across cultures. These face expression categories include happy, fear, anger, surprise, grief, and sadness.

A related approach is to identify a few clusters of variables or factors that result from multidimensional analysis of, for example, questionnaires, and to identify these factors as basic emotions. (Multidimensional analyses such as factor analysis seek to identify a few underlying sources of variance to which a large number of data values such as answers to questions are related.) In one approach, one dimension is associated with valence (positive or negative), and a second dimension is associated with activation or arousal [244,245,246]. In Rolls’ theory of emotion, positive valence might be produced by a reward or by not receiving an expected punisher; and negative valence might be produced by a punisher or by not receiving an expected reward (see Figure 1). Rolls’ theory thus provides a much more detailed analysis of how different reinforcement contingencies can lead to different emotions, especially as each axis in Figure 1 can be produced by different reinforcers, each of which produces different emotions, as set out in Section 2.1. Moreover, I propose that arousal per se is not a dimension of emotion but may be produced as a result of different intensities of reinforcers, moving away from the centre of Figure 1.

One potential problem with some of these approaches is that they risk finding seven plus or minus two categories, which is the maximal number of categories with which humans normally operate, as described in a famous paper by George Miller [247]. A second problem is that there is no special reason why the first few factors (which account for most of the variance) in a factor analysis should provide a complete or principled classification of different emotions or of their functions. In contrast, the theory described here does produce a principled classification of different emotions based on reinforcement contingencies, the nature of the primary and secondary reinforcers, etc., as set out by Rolls [6]. Moreover, the present theory links the functions of emotions to the classification produced by showing how the functions of emotion can be understood in terms of the gene-specified reinforcers that produce different emotions [6,7].

An opposite approach to the dimensional or categorical approach is to attempt to describe the richness of every emotion [248]. Although it is important to understand the richness of every emotion, I believe that this is better performed with a set of underlying principles of the type set out above and by Rolls [6], rather than without any obvious principles to approach the subtlety of emotions.

LeDoux has described a theory of the neural basis of emotion [26,38,116,249] that is probably conceptually similar to that of Rolls [1,2,5,7,70] except that he focuses mostly on classical conditioning which does not require an intervening emotional state, so it is not a well-founded approach to emotion, as stimulus–response learning is involved; except that he focusses on the role of the amygdala in emotion (and not on other brain regions such as the orbitofrontal cortex, which are poorly developed in the rat); except that he focuses mainly on fear (based on his studies of the role of the amygdala and related structures in fear classical conditioning in the rat); and except that he suggests from his neurophysiological findings that an important route for conditioned emotional stimuli to influence behaviour is via the subcortical inputs (especially auditory from the medial part of the medial geniculate nucleus of the thalamus) to the amygdala. In contrast, I suggest that cortical processing to the object representation level before the representation is then sent to areas such as the amygdala and orbitofrontal cortex is normally involved in emotion, as emotions normally occur to objects, faces, etc., and not to spots of light or pure tones, which is what are represented precortically. Further, LeDoux [38] has emphasized especially reflexes and classically conditioned reflexes such as autonomic responses and freezing, which I argue have adaptive value or in LeDoux’s words ‘survival value’, whereas Rolls’ theory is that emotional and motivational states are important intervening states in relation to instrumental actions. The way in which the rodent and amygdala literature has focussed on conditioned responses and not on emotional feelings is described by LeDoux and colleagues [33,34,35,125,250]. Indeed, in this more recent research, LeDoux has reached the view that in humans, the amygdala is not closely involved in emotional feelings, but does not suggest an alternative brain system that is involved in emotion including emotional feelings. I in contrast propose that the key parts of the human brain involved in emotional feelings are the orbitofrontal and anterior cingulate cortices, with the evidence provided above and elsewhere [6,7,8,12,52,61].

Barrett proposed a theory of constructed emotion [251] in which “the dynamics of the default mode, salience and frontoparietal control networks form the computational core of a brain’s dynamic internal working model of the body in the world, entraining sensory and motor systems to create multi-sensory representations of the world at various time scales from the perspective of someone who has a body, all in the service of allostasis.” “In other words, allostasis (predictively regulating the internal milieu) and interoception (representing the internal milieu) are at the anatomical and functional core of the nervous system (see also [252]).” Emotion is held to be constructed from ‘core affect’ (reflecting interoception, e.g., heart rate and energy balance) and ‘context’. The emphasis on interoception is rather at odds with the evidence considered above in Section 6.1 on the James–Lange theory. Rolls’ theory is that many physiological processes involve regulation and, where useful, prediction (an example of which in Rolls’ theory is that a conditioned stimulus such as the sight of food predicts the taste of the food), but emotion is an intervening state produced by a reinforcing stimulus that provides the goals for instrumental behavioural actions required to obtain rewards and avoid punishers. Rewards and punishers may be useful for homeostasis and ‘allostasis’, but Barrett does not base her theory on rewards and punishers and the intervening states required while a goal is being sought by an instrumental action. Nor does Barrett appear to offer any systematic account for all the different emotions that can be produced that depend in Rolls’ theory on the reinforcement contingency, the particular primary reinforcer, the particular secondary reinforcer, the particular combination of reinforcers, the intensity of each reinforcer, what actions are possible, etc., as specified above in Section 2.1 [6,7,8].

Other approaches to emotion are summarized by Keltner et al. [216].

## 7. Comparison with Other Theories of Motivation

### 7.1. Bindra’s Approach to Emotion and Motivation

Bindra [253] offered a “unified interpretation of emotion and motivation”. He drew attention to the fact that motivation is linked “as much to environmental incentive stimuli as to internal organismic conditions”. He wrote in 1969 that “emotions are thought to be provoked externally, while motivation is thought to be generated internally”. By drawing attention to “environmental incentive stimuli” as being important in both motivation and emotion, he sought a unification. He was drawing attention to the fact well known at the time that internal states such as hunger or water depletion increase the reward value of stimuli such as food or water. Thus, externally sensed rewards are important in motivation and also in emotion. He also notes that both emotion and motivation promote actions. He proposes that emotions as well as motivation depend on a central motive state which “arises from an interaction of physiological state and incentive stimuli” [253].

An emphasis on the roles of the reward value of stimuli in motivation was also provided by Rolls [254] “To regulate food intake it appears that the reward value of food is adjusted by the level of hunger, so that a hungry animal eats because it finds food rewarding, and a satiated animal does not eat because it finds food is aversive”. Rolls also emphasized that the hunger signals modulate the reward value of sensory stimuli such as the sight and taste of food, and that the reduction in hunger per se provides very little reward [254]. Concepts such as these led to the development of Rolls’ theory of motivation as described in Section 7.2, as well as to his theory of motivation as described in Section 4.

### 7.2. The Development of Rolls’ Theory of Motivation

#### 7.2.1. Internal Motivational States

In a whole series of studies, we were able to show that internal motivational states such as hunger modulate the reward value of rewarding stimuli such as food, leading to my theory that normally wanting, e.g., hunger, modulates the reward value of food. When hungry, the food is rewarding. Thus, my view is that normally, wanting modulates liking, and that is how internal motivational states such as hunger operate. The development of these ideas follows next.

From early studies of brain-stimulation reward in the monkey, we knew that the reward at some brain sites, such as the macaque orbitofrontal cortex, can be hunger-dependent, with more self-stimulation when hunger was present [255]. The implication is that the state of hunger can modulate the reward value of stimuli, and that the brain-stimulation reward was acting like food reward for a hungry animal.

When we recorded in the macaque lateral hypothalamus and orbitofrontal cortex, we discovered that neurons activated by brain-stimulation reward could in many cases be activated by food reward [256], confirming that the brain-stimulation reward in the experiments just described was mimicking effects of food reward.

When we analyzed the responses of primate lateral hypothalamic neurons to food, we found that they were activated only when hunger was present, and motivated behaviour only occurred for the food reward when hunger was present in experiments in which the individual was fed to satiety with food [257]. We discovered neurons in the orbitofrontal cortex that responded to the taste or sight of food [79] and showed that they also responded to food only when hunger was present in experiments in which the individual was fed to satiety with food [77]. The clear implication is that hunger, an internal motivational state, modulates the reward value of the sight and taste of food. Thus, wanting is related to the reward value and to the liking, in this case, of food.

More direct evidence on the relation between wanting (motivation) and liking (reward value) was found in experiments that were stimulated by our discovery of sensory-specific satiety by neurophysiological experiments in primates, in which we discovered that after eating one food to satiety, such as glucose, neurons would still respond to another food reward, such as peanuts [76]. This discovery was extended to orbitofrontal cortex neurons that respond to the taste [258] or sight of food [77]. The fundamental conceptual point here is that it is not only internal hunger signals related to gastric distension, duodenal stimulation by food, or post-absorptive signals such as rising glucose and insulin concentrations that affect motivational state, but also sensory signals specific to the particular taste or sight of the reward that influence motivation. And it was motivation or wanting that was measured in these experiments in that the primates would only reach out for the food before satiety was produced, and would not reach out for food after satiety was reached. These investigations established a clear link such that high motivation or wanting leads to high reward value of a sensory stimulus, and low motivation leads to low reward value. In this sense, wanting modulates liking.

In closely related investigations in humans, we measured liking by subjective pleasantness ratings and showed directly in this sense that subjective liking was related to hunger, and as the humans were fed to sensory-specific satiety, the rated pleasantness (subjective liking) fell to zero or even a little negative [259,260]. Moreover, to emphasize the importance of sensory-specific satiety as a motivational variable, when humans were unexpectedly offered a second course, a different food to that eaten to satiety was found to be subjectively pleasant (i.e., had high liking) and was associated with eating more of the different food (wanting) [162]. Thus, higher motivation or wanting, as evidenced by wanting to eat more, was associated with higher liking (or pleasantness or reward value).

We then showed that the liking of a food, measured directly by its subjective pleasantness in humans, was correlated with the activation of the medial orbitofrontal cortex, and both the liking and activation of the orbitofrontal cortex fell to zero when the humans were fed to satiety [89]. This again showed that motivation, such as the state of hunger, directly influences the pleasantness or reward value of a stimulus such as food.

All of this evidence shows that motivation, such as a state of hunger, operates by influencing the reward value of food. Thus, motivation (wanting) gates or modulates the reward value and pleasantness of a stimulus (liking), and that is how I think motivation (wanting) is normally related to liking.

Some have argued that drive reduction implements reward (Hull). But I have argued that the above statement is incorrect [5]. The evidence is that a tiny taste on the tongue of a food (i.e., a sensory stimulus) can be a reward to a macaque, rat, or human, but the stomach has to be almost filled with food (to reduce drive) before any reward can be measured [261,262]. And consistently, humans report little reward or pleasure when food is delivered directly into the stomach without any oral taste stimulus, though hunger can be reduced [263].

Corresponding evidence is available that the motivation of thirst signalled by fluid depletion modulates the reward value of the taste of water and does this by modulating reward value representations in the human orbitofrontal cortex of the subjective pleasantness of the taste of water [133,146,264,265].

Thus, Rolls’ theory is that normally, in primates including humans, motivational states related to internal states operate by influencing the reward value and subjective pleasantness in humans of relevant rewards or goals for action, such as the sight and taste of food. In this sense, the motivational state (wanting) modulates liking to implement behavioural choice.

#### 7.2.2. Motivational States Elicited by Emotional States That Are Elicited by External Stimuli

In my theory of emotion as described above, first, an emotional state is produced by an external stimulus such as the sight (or thought of a remembered external stimulus) of a person, and that is a reward that may make us feel happy, but the emotional state is also motivating in that it may also make us want to travel to meet the person. Another example is that if an expected reward is not received, this can have motivating properties to make the individual try in a different way to obtain the reward. Thus, emotion in the first stage of two-factor theory can represent the reward value of a stimulus and produce an emotional state, which can have motivating properties. This is what is represented in the primate including the human orbitofrontal cortex. In the second stage of two-factor theory, we can use instrumental action–outcome learning to learn an action to obtain the reward.

### 7.3. Wanting vs. Liking

In some cases, there can be some departure from what I consider the norm, that wanting normally operates by influencing reward value. These cases, at least often, involve artificial interference of normal operation by influencing dopamine systems in the brain, including in some pathological conditions that may relate to dopamine such as addiction to psychomotor stimulants such as amphetamine and cocaine.

One case is dopamine-implemented stimulation of the mesolimbic dopamine system to the nucleus accumbens/ventral striatum, which can increase the wanting for stimuli, but not the liking for the stimuli [266,267]. Conversely, interfering with this dopamine system can reduce wanting but not liking [266,267]. For example, the blockade of human dopamine receptors by antagonist drugs or dietary-induced reductions in dopamine release did not reduce people’s subjective liking ratings for food pleasure or for drug pleasures of cocaine or heroin, but did reduce people’s subjective reports of wanting to consume more of the drug or food reward [267]. What is termed the incentive salience of the conditioned stimuli can be increased by dopaminergic stimulation.

This may be relevant to conditions when the system is not operating normally, for example in addiction. Addictive drugs, when taken repeatedly in a binge-like fashion by susceptible individuals, can induce long-lasting hyper-reactivity in mesolimbic dopamine-related systems. The sensitized mesolimbic dopamine neurons release more dopamine when a drug is taken, and their dopamine-receiving target neurons become more receptive to excitatory glutamate signals, etc. This may make humans want more of the drug, as in craving, even though the liking or pleasantness of the drug is not increased [163,164,165,266,267,268].

Overall, my view remains as in Section 7.2 that normally, motivation modulates liking or reward value to implement actions to obtain the rewards (or avoid the punishers), but that interference in the normal operation of the system as in some types of addiction may weaken this link that is the typical mode of operation of motivation systems.

### 7.4. Emotion as Wanting

In his book *Understanding Motivation and Emotion*, Reeve [269] defines emotion as *wanting*. He defines emotion as “complex but coordinated feeling-arousal-purposive-expressive reactions to the significant events in our lives (e.g., an opportunity, a threat, and a loss)”. He continues “emotions rapidly and rather automatically generate and synchronize four interrelated aspects of experience into a unified whole: *Feelings*: subjective, verbal, descriptions of emotional experience. *Arousal*: Bodily mobilization to deal with situational demands. *Purpose*: Motivational urge to accomplish something specific at the moment. *Expression*: Nonverbal communication of our emotional experience to others.” He regards emotion as a subset of motivation. He also writes the following [269]: “Motivation is a private and unobservable (internal) experience”.

That type of approach to motivation and emotion [269] (in contrast to Rolls’s theory of emotion and motivation) does not lead to any theory of emotion and motivation that can apply to others than humans; does not clearly operationalize emotion as, for example, states elicited by rewards and punishers and motivations as states elicited when rewards are being sought (i.e., before they are obtained) or punishers; does not lead to a systematic treatment of how different emotions are produced (see Section 2.1); and is not set firmly in the supporting context of our understanding of the brain mechanisms of emotion and motivation [5,6,7,8,10,11,12,72].

### 7.5. Evaluation

Rolls’ theory of motivation goes beyond some earlier approaches by showing how motivation can be related in some cases to internal states such as hunger and thirst which modulate reward value (subjective liking), and can be related to emotion produced by external stimuli, which can have motivational effects; how the same unlearned rewards and punishers have evolved by gene selection to be useful for both emotion and motivation by specifying outcomes of rewards and punishers (emotions), as well as the anticipation of rewards and punishers (motivations); and can be closely related to the underlying brain mechanisms of motivation in both humans and other animals [5,6,7,8,10,11,12,72].

## 8. Conclusions and Highlights

1. A new approach is further developed here to produce a unified understanding of emotion and motivation, with the underlying brain mechanisms considered in more detail elsewhere [6,7,8,12] but summarized next. In this unified theory of emotion and motivation, motivational states are states in which instrumental goal-directed actions are performed to obtain rewards or avoid punishers, and emotional states are states that are elicited when the reward or punisher is or is not received. This greatly simplifies our understanding of emotion and motivation, for the same set of genes and associated brain systems can define the primary or unlearned rewards and punishers such as a sweet taste or pain that can be used for both emotion and motivation. A very wide range of emotions can be understood by taking into account the reinforcement contingency, the particular primary reinforcer, the particular secondary reinforcer, the particular combination of reinforcers, the intensity of each reinforcer, etc., as specified above in Section 2.1 [6,7,8].
2. New evidence on the connectivity in humans of brain systems involved in emotion and motivation is available from measures of the effective connectivity between 360 cortical regions in the Human Connectome Project MultiModal Parcellation atlas (HCP-MMP) [65] and is complemented by the addition of 66 subcortical regions [66]. The cortical regions in this atlas are defined by anatomical characteristics (cortical myelin content and cortical thickness), functional connectivity, and task-related fMRI and provide a useful basis for understanding brain regions with different connectivity and potentially different computational functions. Some of the following points reflect advances in our understanding of brain systems involved in emotion by taking into account the effective connectivity of the human brain, complemented by functional connectivity and diffusion tractography [20,61,67,68,184,213,270,271].
3. It is shown that the primate including the human orbitofrontal cortex represents primary reinforcers such as taste, pain, and pleasant touch, with this information reaching the orbitofrontal cortex from the primary taste cortex in the anterior insula and from somatosensory cortical regions. It is shown that the primate including the human orbitofrontal cortex learns associations between these primary reinforcers and secondary reinforcers such as the sight of food or of an aversive stimulus in one trial and can reverse these associations in one trial using a rule- or model-based computation model. These stimulus–stimulus learned representations are of expected value (and can be thought of as predictions). The representations in the orbitofrontal cortex are value based and are appropriate for being the goals for motivated behaviour and for eliciting emotional states. Actions are not represented in the primate orbitofrontal cortex. Other inputs to the orbitofrontal cortex are about socially relevant stimuli such as face expression and face identity and relate to inputs from the cortex in the superior temporal sulcus. Rewards tend to be represented in the human medial orbitofrontal cortex and punishers and non-reward in the lateral orbitofrontal cortex. This evidence is complemented by the effects of damage to the orbitofrontal cortex in humans, which impairs reward-related reversal learning, emotional responses, and subjective emotional feelings. In primates including humans, reward and punisher value (the ‘valence’ of stimuli) is not represented in cortical stages of sensory processing prior to the orbitofrontal cortex, such as the insular primary taste cortex and inferior temporal visual cortex.
4. The ventromedial prefrontal cortex (vmPFC), which receives signals from the orbitofrontal cortex, is activated by rewards, is implicated in reward-related decision-making, and has connectivity to the pregenual and supracallosal anterior cingulate cortices [20] (Figure 4).
5. The human medial and lateral orbitofrontal cortex and the vmPFC, have connectivity to the pregenual anterior cingulate cortex, which is strongly activated by rewards and which projects to the hippocampal system, both directly and via the posterior cingulate cortex (Figure 4) [20]. It is proposed that this provides the route for rewards and emotional states to become part of episodic memory. It is further proposed that the reward/emotional value of recalled episodic memories is important in influencing which memories are further processed and become incorporated into long-term semantic memory. It is further proposed that this route enables goals for navigation to enter the human hippocampal system, and indeed, navigation is almost always to obtain goals, which are reflected in hippocampal neuronal activity [272,273].
6. The human pregenual anterior cingulate cortex has effective connectivity to the septum, from which cholinergic neurons important in memory consolidation project to the hippocampus (Figure 4) [20]. The human medial orbitofrontal cortex (region pOFC) has effective connectivity to the basal forebrain magnocellular nucleus of Meynert, from which cholinergic neurons important in memory consolidation project to the hippocampus (Figure 4) [20]. It is proposed that through these routes, the value system can influence memory consolidation. Consistent with this, damage to the vmPFC/anterior cingulate cortex in humans impairs memory. It is argued that the human orbitofrontal cortex/vmPFC/pregenual anterior cingulate cortex is not a memory system, but a value system, and that this value/emotion system influences memory and memory consolidation through these connectivities [64].
7. The orbitofrontal cortex and pregenual anterior cingulate cortex have connectivity in humans to the supracallosal anterior cingulate cortex, which in turn has connectivity to premotor cortical regions including the midcingulate premotor cortex. It is proposed that these routes provide for action–outcome learning in the supracallosal anterior cingulate cortex, where the outcome is the reward or punisher received from the orbitofrontal cortex and pregenual anterior cingulate cortex [8,12].
8. With this foundation, it is proposed that the function of the primate orbitofrontal cortex in emotion is to represent rewards and punishers and to implement stimulus–reward/punisher association learning and reversal (i.e., stimulus–stimulus learning). It is argued that in contrast, the role of the supracallosal anterior cingulate cortex is to learn associations between actions and the rewards/punishers that follow the actions, and with this action–outcome learning to influence the future choice of actions when reward/aversive expected value stimuli are received from the orbitofrontal cortex.
9. It is shown that the human amygdala has effective connectivity from relatively few cortical regions, primarily those in the anterior temporal lobe, and even less effective connectivity back to the neocortex. The outputs of the human amygdala are directed primarily to brainstem regions involved in autonomic responses, cortical arousal, and some behavioural responses. In line with this, there is evidence that the human amygdala is much less involved in reported, experienced, declarative, emotion than the orbitofrontal cortex. This is a key re-evaluation of the functions of the human amygdala in emotion [61].
10. It is shown that in addition to these emotion-related outputs to behaviour, in humans and perhaps in other animals, there is a rational, reasoning route to action that may override the genes selected during evolution to specify the rewards and punishers important in the control of goal-directed behaviour. The reasoning route to action may make choices in the interests of the individual and the phenotype, not in the interests of the gene-specified rewards, but it could also optimize the actions taken to obtain gene-specified rewards.
11. Damage to the orbitofrontal cortex in humans can produce neurological changes such as reduced ability to respond correctly to emotion-relevant stimuli such as face and voice expression and to learn and change behaviour in response to reinforcement contingencies. It is shown that altered connectivity of the orbitofrontal cortex with other brain regions, and the sensitivity of the medial orbitofrontal cortex to rewards and of the lateral orbitofrontal cortex to punishers, is involved in human depression. Damage to the human orbitofrontal cortex, and much less to the amygdala, impairs human subjective emotional experiences and feelings.

## Figures and Tables

**Table 1:** Some primary reinforcers and the dimensions of the environment to which they are tuned.

**Taste**	
Salt taste	reward in salt deficiency
Sweet	reward in energy deficiency
Bitter	punisher, an indicator of possible poison
Sour	punisher
Umami	reward, an indicator of protein; produced by monosodium glutamate and inosine monophosphate
Tannic acid	punisher; it prevents absorption of protein; found in old leaves; probably somatosensory not gustatory (Critchley and Rolls 1996 [17])
**Odor**	
Putrefying odor	Punisher, a hazard to health
Pheromones	reward (depending on the hormonal state)
**Somatosensory**	
Pain	punisher
Touch	reward
Grooming	reward; to give grooming may also be a primary reinforcer
Washing	reward
Temperature	reward if it tends to help maintain normal body temperature; otherwise, a punisher
**Visual**	
Snakes, etc.	punisher for, e.g., primates
Youthfulness	reward, associated with mate choice
Beauty, e.g., symmetry	reward
Secondary sexual characteristics	rewards
Face expression	reward (e.g., smile) or punisher (e.g., threat)
Blue sky, cover, open space	reward, indicator of safety
Flowers	reward (indicator of fruit later in the season?)
**Auditory**	
Warning call	punisher
Aggressive vocalization	punisher
Soothing vocalization	reward (part of the evolutionary history of music, which at least in its origins taps into the channels used for the communication of emotions)
**Reproduction**	
Courtship	reward
Sexual behavior	reward (different reinforcers, including a low waist-to-hip ratio, and attractiveness influenced by symmetry and being found attractive by members of the other sex).
Mate guarding	reward for a male to protect his parental investment.
	Jealousy results if his mate is courted by another male,
	because this may ruin his parental investment
Nest building	reward (when expecting young)
Parental attachment (love)	reward (good for the parent’s genes
	both when the attachment is to the other parent or an infant)
Infant attachment to parents (love) Crying of infant	reward (good for the infant’s genes) punisher to parents; produced to promote successful development
Power, status, wealth, resources	Attractive to females, who may benefit from resources for their offspring.
	Attractive to males as they make males attractive to females.
Body size	Large in males may be attractive to females as a signal
	for the provision of protection
	and of the ability of her male offspring to compete for a mate.
	Small in females may be attractive to males as a neotenous sign
	of youth, and therefore fertility
**Other**	
Novel stimuli	rewards (encourage animals to investigate the full possibilities
	of the multidimensional space in which their genes are operating)
Sleep	reward; minimizes nutritional requirements and protects from danger
Altruism to genetic kin	reward (kin altruism)
Altruism to other individuals	reward while the altruism is reciprocated
	in a ‘tit-for-tat’ reciprocation (reciprocal altruism).
	Forgiveness, honesty, and altruistic punishment
	are some associated heuristics.
	May provide underpinning for some aspects of what is felt to be moral.
	punisher when the altruism is not reciprocated
Group acceptance, reputation Control over actions	reward (social greeting might indicate this). These goals can account for why some cultural goals are pursued reward
Play	reward
Danger, stimulation, excitement	reward if not too extreme (adaptive because of practice?)
Exercise	reward (keeps the body fit for action)
Mind reading	reward; practice in reading others’ minds, which might be adaptive
Solving an intellectual problem	reward (practice in which might be adaptive)
Storing, collecting	reward (e.g., food)
Habitat preference, home, territory	reward
Some responses	reward (e.g., pecking in chickens, pigeons;
	adaptive because it is a simple way in which eating grain
	can be programmed for a relatively fixed type of environmental stimulus)

## Data Availability

No new data were analyzed for this review paper. The effective connectivity, functional connectivity, and diffusion tractography analyses referred to here [20,61,67,68,184,213,270,271,274,275,276] were performed with Human Connectome Project data [277], which are available at the HCP website http://www.humanconnectome.org/ (accessed on 12 March 2025). The Human Connectome Project Multimodal Parcellation atlas and its availability are described by [65], and the extended version HCPex is described by Huang and Rolls et al. [66] and is available at https://www.oxcns.org (accessed on 12 March 2025).
